# Chemiluminescence-initiated and *in situ*-enhanced photoisomerization for tissue-depth-independent photo-controlled drug release†
†Electronic supplementary information (ESI) available. See DOI: 10.1039/c8sc04012e


**DOI:** 10.1039/c8sc04012e

**Published:** 2018-11-10

**Authors:** Yufu Tang, Xiaomei Lu, Chao Yin, Hui Zhao, Wenbo Hu, Xiaoming Hu, Yuanyuan Li, Zhen Yang, Feng Lu, Quli Fan, Wei Huang

**Affiliations:** a Key Laboratory for Organic Electronics and Information Displays , Jiangsu Key Laboratory for Biosensors , Institute of Advanced Materials (IAM) , Jiangsu National Synergetic Innovation Center for Advanced Materials (SICAM) , Nanjing University of Posts & Telecommunications (NUPT) , Nanjing 210023 , China . Email: iamqlfan@njupt.edu.cn; b Key Laboratory of Flexible Electronics (KLOFE) , Institute of Advanced Materials (IAM) , Jiangsu National Synergetic Innovation Center for Advanced Materials (SICAM) , Nanjing Tech University (Nanjing Tech) , Nanjing 211816 , China; c Shaanxi Institute of Flexible Electronics (SIFE) , Northwestern Polytechnical University (NPU) , Xi'an 710072 , China

## Abstract

Tissue-penetration-depth-independent self-luminescence is highly expected to perform photoisomerization-related bioapplications *in vivo* to overcome the limitation of shallow tissue-penetration from external photoexcitation. However, it remains extremely challenging because of lacking a target-specific high-intensity self-luminescence to precisely and effectively drive the photoisomerization. Here, we first report a target-specific tissue-depth-independent photoisomerization *in vivo* by developing a target-specific initiated and *in situ*-enhanced chemiluminescence (one of self-luminescence) strategy that overcomes the limitation of lacking target-specific high-intensity self-luminescence. Considering that photoisomerization shows boundless glamour in drug-controlled release for disease-specific chemotherapy, we demonstrated applicability of our strategy to apply it in tumor-specific self-luminescence-controlled drug chemotherapy. Specifically, a chemiluminescence substrate and chemiluminescence fluorophore (antitumor drug, CPT) were co-encapsulated in host–guest carriers composed of cyclodextrin and the photoisomerization molecule azobenzene. Tumor-specific H_2_O_2_-induced chemiluminescence preliminarily isomerizes azobenzene, triggering the partial dissociation of host–guest carriers and CPT release. Particularly, the initially released CPT again functions as a chemiluminescence enhancer to achieve *in situ* enhanced chemiluminescence, assuring target-specific enhanced isomerization and CPT release. With high tumor-inhibition-rate (73%) and no obvious therapy-side-effect *in vivo* indicates the good efficiency and target-specificity of our chemiluminescence-driven photoisomerization. Although we only demonstrated one example of a photoisomerization-related bioapplication, namely photoisomerization-controlled drug chemotherapy, our work provides guidelines to design various target-specific tissue-depth-independent photoisomerization for bioapplications.

## Abstract 2

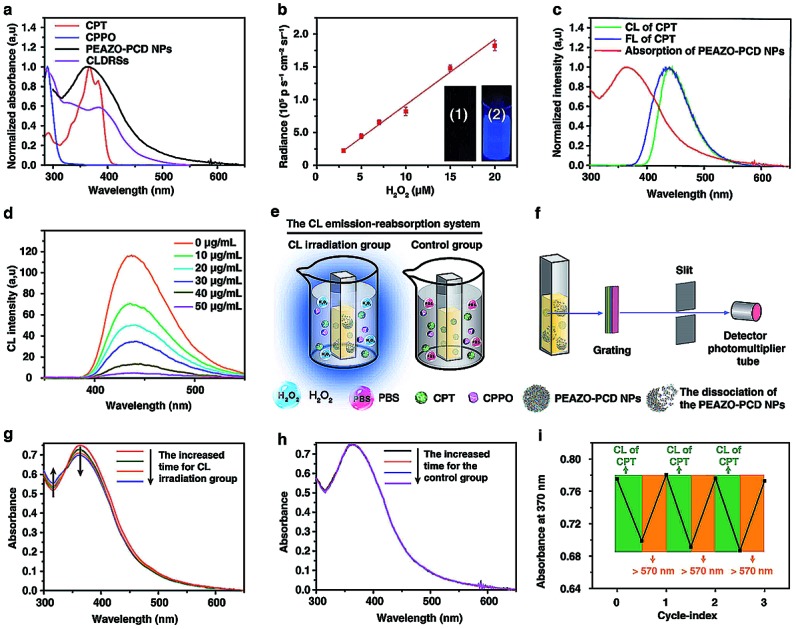
Tissue-penetration-depth-independent self-luminescence is highly expected to perform photoisomerization-related bioapplications *in vivo* to overcome the limitation of shallow tissue-penetration from external photoexcitation.

## Introduction

Photoisomerization that can spatiotemporally change structure between isomers upon photoexcitation is one of the most promising strategies for precisely controlling biological structures/function in a living body.^1^ This has been widely studied in biomedicine, including photo-controlled drug release,^2^–^4^ optogenetics,^5^ and optopharmacology.^6^ Unfortunately, all these applications *in vivo* are severely limited to shallow tissue because most photoisomerization materials can only be excited by UV/Vis light which has poor tissue penetration.^7^,^8^ Recently, the use of near-infrared light with longer wavelength (650–1000 nm) through upconversion, such as two-photon,^4^,^5^ upconverting nanoparticles,^2^ and triplet–triplet annihilation,^9^ is proposed to improve tissue penetration, but it only increases to millimetre depths. Self-luminescence, including chemiluminescence (CL),^10^–^23^ bioluminescence,^24^–^27^ and Cerenkov radiation,^28^ are now deemed to have more potential as tissue-penetration-depth-independent light and has been successfully applied in the highly sensitive bioimaging field.^8^ However, self-luminescence-driven photoisomerization *in vivo* remains extremely challenging because such a low-intensity light source insufficiently implements a good photoisomerization effect.^8^ Thus, developing a self-luminescence source that inherits an ability of external light to specifically select a target site and also provide sufficient intensity is highly desirable for implementing target-specific tissue-depth-independent photoisomerization for *in vivo* application.

As one of frequently used self-luminescence techniques, CL usually results from a CL substrate rapidly and selectively reacting with hydrogen peroxide (H_2_O_2_) to produce chemical reaction energy for exciting nearby CL fluorophores.^10^–^23^ Since a high H_2_O_2_ level is a feature of many target tissues, for example, the higher H_2_O_2_ level (ranging from 0.1 to 1 mM) in tumors^29^–^31^ than in normal tissues (∼10^–4^ mM),^15^,^32^ it can endow CL with the ability of target-specific initiation. While CL intensity shows rapid recession along with the consumption of H_2_O_2_, and consequently very few high-intensity-light-implemented bioapplications (*e.g.*, photodynamic/photothermally therapy and photochemical bond cleavage) using CL as a light source have been developed except for two recent attempts in tumor photodynamic therapy.^14^,^18^ One employed direct intratumor injection of a CL source and H_2_O_2_ and the other used oral administration of H_2_O_2_ enhancer to realize enhanced CL.^14^,^18^ However, these CL enhancement examples lacked a strategy to precisely amplify the signal at the target site, which is inadequate for implementing target-specific photoisomerization *in vivo*.

**Scheme 1:**
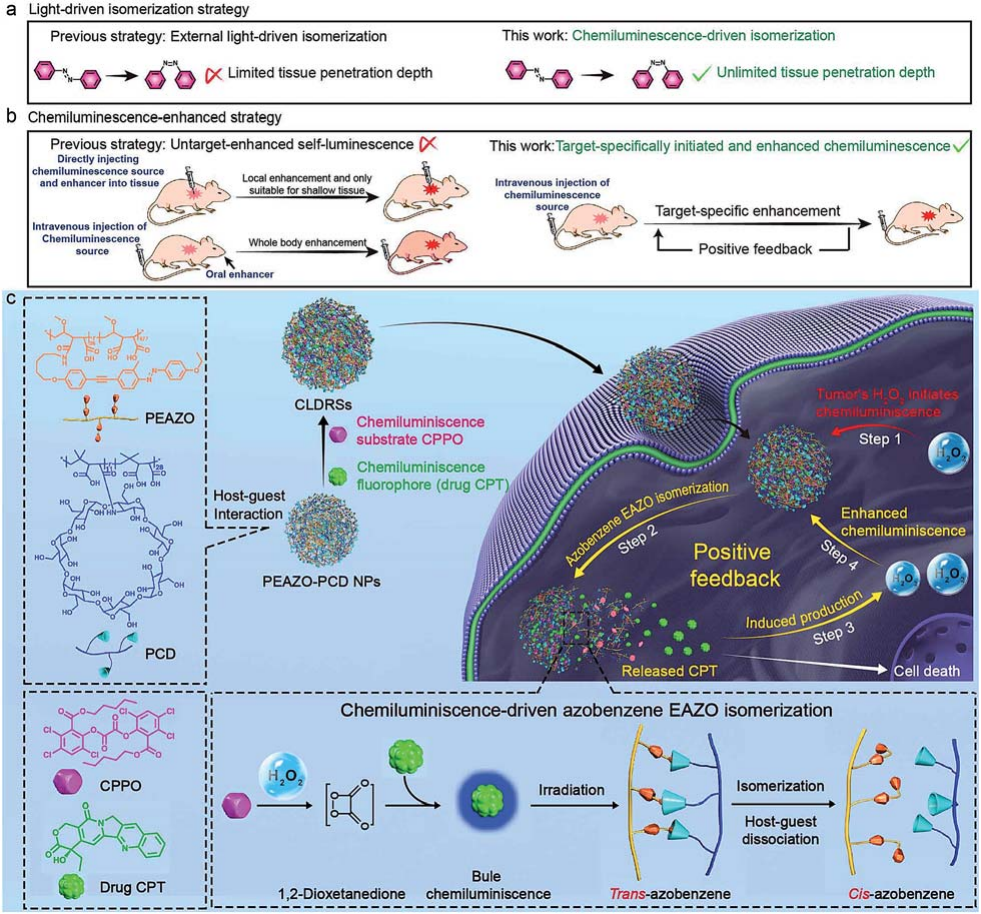
(a and b) Schematic illustration of the differences between previous works and our works. (c) Schematic illustration of the concept of target-specific tissue-depth-independent photoisomerization and one example of this concept bioapplication in the photo-controlled drug release field for tumor chemotherapy.

Herein, we report a precise tissue-depth-independent photoisomerization *in vivo* by developing a target-specifically initiated and *in situ*-enhanced CL strategy that solves restrictions of low self-luminescence intensity and lacking a method to precisely amplify the signal at the target site. Furthermore, considering that photoisomerization-controlled drug release is one of crucial embranchment of photoisomerization-related bioapplications, we first demonstrated the applicability of our strategy to construct target-specific tissue-depth-independent photo-controlled drug release systems (CLDRSs) for tumor chemotherapy. Specifically, the CL substrate peroxyoxalate (CPPO) and CL fluorophore (antitumor drug camptothecin, CPT) were used as two building units to construct the CL source. Host–guest nanoparticles (PEAZO-PCD NPs) self-assembled from a photoisomerization molecule, azobenzene (EAZO)-pendant polymer (PEAZO) and a cyclodextrin-pendent polymer (PCD), served as the carrier to encapsulate CPPO and CPT (Scheme 1). In a tumor, the EAZO groups can preliminarily be isomerized by the existing-H_2_O_2_-induced CL from the drug CPT, thus triggering partial dissociation of the host–guest carrier and release of the drug CPT. Subsequently, the initially released CPT again functions as a H_2_O_2_ enhancer to induce a higher H_2_O_2_ level in a tumor. Such a positive-feedback-mechanism-improved the H_2_O_2_ level and, in turn, promoted enhanced CL (*in situ*-enhanced CL) of subsequently accumulative CLDRSs and incompletely dissociative CLDRSs in tumors to realize enhanced EAZO photoisomerization and CPT release. In contrast, a low-level-H_2_O_2_-induced weak CL in normal tissues which barely initiated the isomerization, not to mention an enhanced isomerization effect. Such target-specific initiated and self-enhanced CL by a positive feedback mechanism endowed CL with the ability to precisely accomplish EAZO isomerization and drug CPT release. High tumor-inhibition-rate (73%) and no obvious therapy-side-effect *in vivo* indicated good efficiency and target-specificity of our chemiluminescence-driven photoisomerization. Considering that many diseases have the feature of high level H_2_O_2_ and that CPT can easily be replaced by other UV/Vis fluorophores or H_2_O_2_ enhancers (*e.g.*, vitamin C), thus, although we only demonstrated one example of target-specific self-luminescence-driven photoisomerization in a bioapplication, namely photoisomerization for drug-controlled release, our work provides guidelines to design various other photoisomerization-related biomedical applications with target-specificity and a depth-independent manner *in vivo*.

## Results and discussion

**Fig. 1:**
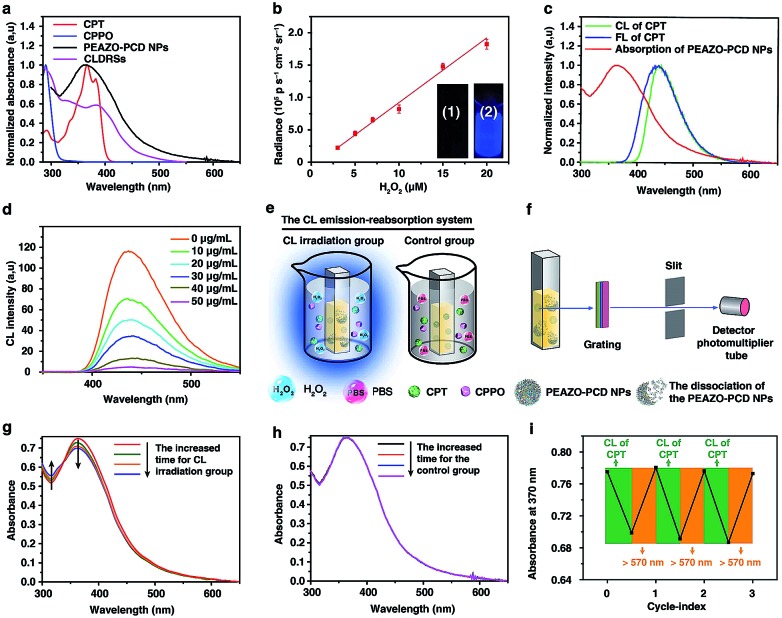
Photoisomerization of the EAZO moieties induced by CL. (a) Normalized absorption spectra of CPT and CPPO in a 1 : 9 DMF–PBS solution, PEAZO-PCD NPs and CLDRSs in PBS. (b) CL intensities of CPT (5 μg mL^–1^, 1 mL), CPPO (5 μg mL^–1^, 1 mL) and PCD (10 μg mL^–1^, 1 mL) as a function of H_2_O_2_ concentration in a 1 : 9 DMF–PBS solution. The inset is a photograph of the before (1) and after (2) generated CL in the dark, taken at 10 s after addition of H_2_O_2_ (0.05 M) with error bars and standard deviation (s.d.) (*n* = 3 replicates). (c) Normalized absorption of PEAZO-PCD NPs in PBS (pH = 7.4) and fluorescence (FL) and CL spectra of CPT in a 1 : 9 DMF–PBS solution. (d) The CL emission intensity of CPT which vanishes when the PEAZO-PCD NPs increases. (e) Schematic layout of the emission–reabsorption system. (f) Schematic layout of the change in the absorption spectra of EAZO moieties measurement system with a spectrophotometer containing the photomultiplier tube. Change in the absorption spectra of the PBS solution of PEAZO-PCD NPs (g) with and (h) without the CL irradiation for different times. (i) Multiple rounds of *cis*–*trans* isomerization of EAZO with alternating CL of CPT and external yellow light (>570 nm) irradiation in PBS solution.

EAZO, (4-ethoxy-2′-methyl-4′-(6-hydroxyhexyloxyphenylethynyl)-azobenzene), can be easily isomerized within a few seconds under low-intensity sunlight or ultralow-power external UV-Vis light (even as low as 1 mW cm^–2^,^9^,^33^ far below the currently reported^34^ minimum light power of ∼10 mW cm^–2^ for photodynamic therapy). Since CL has been successfully used instead of external light or sunlight for photodynamic therapy^14^,^16^–^18^ and plant photosynthesis,^35^ we envisioned that this EAZO might also be isomerized by CL irradiation. PCD and PEAZO (see Scheme 1c) were synthesized using an amidation reaction by conjugation of 3A-amino-3A-deoxy-(2AS,3AS)-β-cyclodextrin hydrate (β-CD-NH_2_) and azobenzene EAZO groups into poly(isobutylene-*alt*-maleic anhydride) (*M*_n_ = 6 kDa) and poly(methyl vinyl ether-*alt*-maleic anhydride) (*M*_n_ = 80 kDa), respectively. The average numbers of EAZO and β-CD-NH_2_ molecules within each PEAZO and PCD chain were about 11 and 36, respectively (see the ESI†). The stability constant (*K*_c_) of β-CD-NH_2_ cyclodextrin with the azobenzene EAZO precursor was measured to be 4 × 10^4^ M^–1^ (Fig. S1†), close to that of cyclodextrin with azobenzene (*trans*, about 10^5^ M^–1^) in previous literature reports.^36^,^37^ Given that it is much higher than the *K*_c_ of cyclodextrin with CPT (266 M^–1^)^3^,^36^,^38^ and CPPO (1890 M^–1^) (Fig. S2†), cyclodextrin preferentially forms inclusion complexes with EAZO in our system, which is consistent with previous reports.^3^,^36^ Then, water-soluble host–guest nanoparticles (PEAZO-PCD NPs, molar ratio of EAZO/β-CD-NH_2_ = 1 : 2) were successfully constructed from PEAZO and PCD using a previously reported method.^39^,^40^ Furthermore, CLDRS (CPT + CPPO@PEAZO-PCD NPs) were prepared by encapsulating hydrophobic CPT and CPPO into the PEAZO-PCD NPs. The UV-Vis spectrum of the CLDRS exhibited the characteristic absorption peaks of CPT and CPPO (Fig. 1a), indicating their successful loading. The loading capacity of CPPO and CPT (if ever present) were 151 and 186 μg mg^–1^, respectively (details in the ESI†). Two other nanoparticles (NPs) simply containing CPT (CPT@PEAZO-PCD NPs) and CPPO (CPPO@PEAZO-PCD NPs) were also prepared using the same method as the controls.

To investigate properties of CL of CPT in CLDRSs, CPT combined with CPPO and PCD (a CPT + CPPO + PCD mixture) was used in the presence of H_2_O_2_ in a 1 : 9 DMF–PBS solution (pH = 7.4). As expected, a blue CL of CPT was observed (Fig. 1b, inset). The CL intensity was proportional to the H_2_O_2_ concentration (Fig. 1b) and ∼900-fold higher as compared to other reactive oxygen species (Fig. S3†), demonstrating that the peroxyoxalate CL system had H_2_O_2_-specific luminescence ability. Also, it showed a long half-life of ∼2 hours and its intensity only decreased by three-fifths even after 3 hours (Fig. S4†). Such long half-life and high intensity may be because cyclodextrin slows down the reaction between CPPO and H_2_O_2_ and improves the luminous efficiency of CPT,^41^ which is helpful for providing light energy to trigger EAZO photoisomerization. It also should be beneficial for *in vivo* applications.^13^,^18^

As shown in Fig. 1c, the fluorescence (FL) spectrum of CPT closely resembled its CL spectrum and matched well with the broad and strong absorption band of EAZO moieties in PEAZO-PCD NPs at 300–600 nm, which provides a great possibility for the emission–reabsorption process between CL emission and azobenzene EAZO moieties absorption. The dramatically decreased CL intensity of CPT at 435 nm after adding PEAZO-PCD NPs confirmed that CL of CPT can be efficiently absorbed by EAZO moieties through the emission–reabsorption process (Fig. 1d). Since this EAZO underwent *trans*–*cis* photoisomerization upon exposure to light at <570 nm and *cis-*EAZO returned to *trans-*forms with light at >570 nm irradiation,^42^,^43^ we envisioned that the CL of CPT was suitable for EAZO isomerization. To confirm whether the CL could realize *trans*–*cis* isomerization of EAZO, we attempted to provided classical evidence of the change in a *trans*–*cis* absorption spectrum of EAZO upon CL irradiation.^2^,^9^ However, because of the severe overlap between the absorption spectra of CPT and PEAZO-PCD NPs (Fig. 1a), we could not directly evaluate the change in *trans*–*cis* absorption spectrum change of EAZO moieties in our CLDRS. Thus, the emission–reabsorption process using an experimental setup (Fig. 1e) as previously reported^9^ was applied for this purpose. The CL source (CPT + CPPO + PCD mixture and 2 mM H_2_O_2_) were filled in a 1 : 9 DMF–PBS solution (pH = 7.4) in a beaker (Fig. 1e, left), and the generated CL was used to irradiate a PBS solution of the PEAZO-PCD NPs in an optically transparent cell. The control group (Fig. 1e, right) used the exact same procedure as the CL irradiation group (Fig. 1e, left) except using the same volume of PBS instead of H_2_O_2_. The changes in *trans*–*cis* absorption spectra of EAZO moieties were then measured with an experimental setup which was analogous to that in Fig. 1f. Upon exposure to the CL of CPT with increasing time, as previously reported,^9^,^43^ the characteristic absorbance of the *trans*-EAZO group at approximately 370 nm obviously decreased, while the peak of the *cis*-EAZO group at 320 nm was gradually elevated (Fig. 1g). By contrast, no absorption change was observed in the control group (Fig. 1h), indicating that the CL of CPT can cause photoisomerization of the EAZO moieties in the PEAZO-PCD NPs. Additionally, multiple rounds of *trans*–*cis* isomerization of EAZO moieties in PEAZO-PCD NPs with alternating CL of CPT and external yellow light (>570 nm) irradiation in PBS solution showed that the CL-irradiation-isomerized *cis*-EAZO can be recovered to the *trans*-EAZO (Fig. 1i), further confirming the successful CL-triggered photoisomerization of the EAZO group. Thanks to the closer distance between CPT and EAZO in CLDRSs (CPT + CPPO@PEAZO-PCD NPs), we believe a much better CL emission–reabsorption process will occur, which can acquire more effective photoisomerization of EAZO moieties in CLDRSs.

**Fig. 2:**
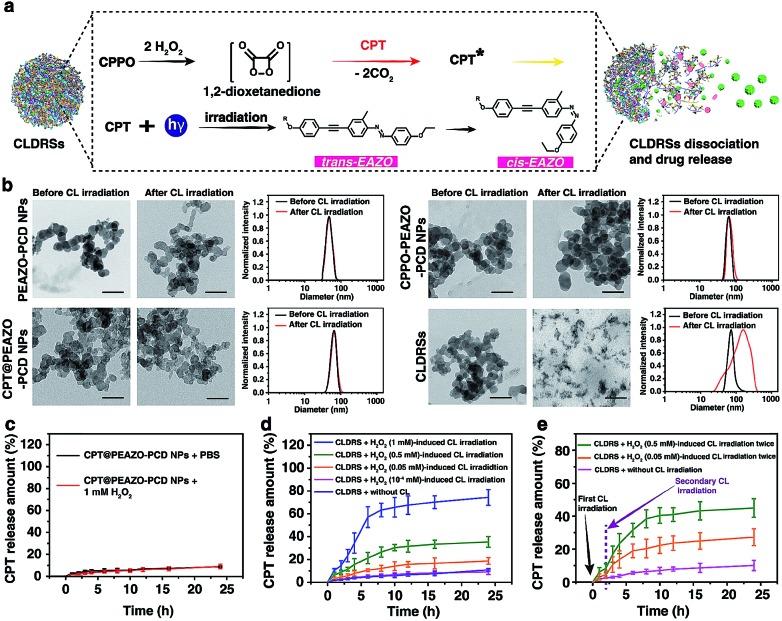
CL-driven photoisomerization for *in vitro* CPT-controlled release from CLDRSs. (a) Illustration of CL-triggered drug release mechanism of CLDRSs. (b) Changes in TEM and DLS of PEAZO-PCD NPs, CPT@PEAZO-PCD NPs, CPPO@PEAZO-PCD NPs, and CLDRSs after CL of CPT irradiation (CL induced by 2 mM H_2_O_2_). Scale bar, 100 nm. (c) Drug release curves of CPT@PEAZO-PCD NPs under H_2_O_2_ (1 mM) treatment. (d) Drug release curves of CLDRSs under different intensity of CL of CPT irradiation. (e) Drug release curves of CLDRSs under CL of CPT irradiation. Secondary CL irradiation indicates addition of 0.05 mM H_2_O_2_ or 0.5 mM H_2_O_2_ to enhance CL at the 16th hour. Error bars indicate the s.d. (*n* = 3).

Fig. 2a shows a classical CL process.^12^,^44^ CPPO rapidly and selectively decomposes in the presence of H_2_O_2_ to form the high-energy 1,2-dioxetanedione intermediate known to excite the nearby fluorescent drug CPT to emit blue CL. The CL subsequently induces EAZO isomerization to cause the dissociation of EAZO from β-CD-NH_2_ cyclodextrin, further triggering the dissociation of CLDRSs and CPT release. To verify the feasibility of CL-driven azobenzene EAZO photoisomerization for CPT release, we first studied the time-dependent stability of the NPs (CPT@PEAZO-PCD NPs, CPPO@PEAZO-PCD NPs and CLDRSs) through DLS analysis and a dialysis experiment (Fig. S5–S8†). These NPs were stable after storage in the dark for 24 hours in PBS and FBS because the supramolecular polymer PEAZO-PCD has a higher stability constant compared with the monovalent complex.^45^ Then, we added H_2_O_2_ into CLDRSs to induce EAZO isomerization for a drug release study. The CLDRSs (CPT + CPPO@PEAZO-PCD NPs) collapsed into irregular aggregates and their hydrodynamic size changed from predominantly 70 nm to a random distribution from H_2_O_2_-induced CL irradiation (Fig. 2b). Comparatively, PEAZO-PCD NPs, CPT@PEAZO-PCD NPs, and CPPO@PEAZO-PCD NPs showed little changed sizes because they cannot generate an effective CL in the presence of H_2_O_2_ to isomerize EAZO (Fig. 2b). These results demonstrated that the CL effectively drove EAZO isomerization in CLDRSs and thus successfully dissociated the CLDRS. Drug release ability was further studied by dialysis of those NPs in PBS (pH = 7.4) under H_2_O_2_-induced CL irradiation. We first added H_2_O_2_ into the CPT@PEAZO-PCD NPs. Drug release amounts showed no obvious differences whether there was H_2_O_2_ or not (Fig. 2c), indicating that H_2_O_2_ itself did not result in drug CPT release without the CL substrate CPPO. For our CLDRSs (CPT + CPPO@PEAZO-PCD NPs), without adding H_2_O_2_, only a relatively small amount of CPT (approximately 10 wt%) was released within 24 h (Fig. 2d). By contrast, significant amounts of CPT were released from CLDRSs, reaching 35 wt% release in 24 h in the presence of H_2_O_2_ (0.5 mM)-induced CL irritation and even 72 wt% release in the presence of H_2_O_2_ (1 mM)-induced CL irritation (Fig. 2d). The 17 wt% release can still be obtained when irradiated by H_2_O_2_ (0.05 mM)-induced CL. However, the drug release was ignorable using H_2_O_2_ (10^–4^ mM)-induced CL irradiation. These results clearly showed that the drug release was caused by CL irradiation and the released amounts are highly correlated with H_2_O_2_-induced CL intensity. Considering the H_2_O_2_ concentration in a tumor (concentrations ranging from 0.1 mM to 1 mM (ref. 29)) was higher than in normal tissues (∼10^–4^ μM),^32^ it may trigger in the tumor environment-specific dissociation of the CLDRS. Interestingly, two hours later following the first H_2_O_2_ (0.5 mM)-induced CL irradiation, an additional 15 wt% CPT was released upon a secondary 0.5 mM H_2_O_2_ (Fig. 2e). Even employing 0.05 mM H_2_O_2_ can produce an additional 6 wt% CPT release (Fig. 2e). All these results (Fig. 1b, 2d and e) indicated that maintaining a high concentration of H_2_O_2_ can effectively achieve high CL-intensity to further enhance azobenzene EAZO photoisomerization, leading to increased drug release.

**Fig. 3:**
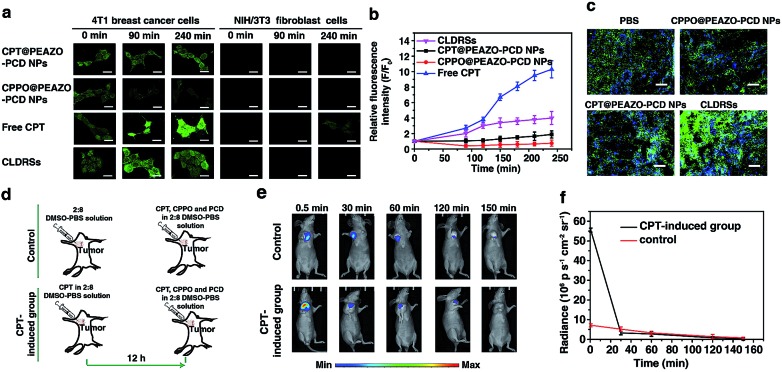
CPT-induced H_2_O_2_ production in tumor cells and tissues to achieve self-enhanced CL. (a) Time-dependent measurements of the H_2_O_2_ level using H_2_O_2_ probe DCFH-DA (10 μM) in 4T1 tumor cells and 3T3 normal cells with different treatments. Scale bar, 30 μm. (b) Quantitatively relative fluorescence intensity of (a) for 4T1 tumor cells. (c) *Ex vivo* H_2_O_2_ generation intravenous injection of various interventions in tumor measured using DCFH-DA (green) and Hoechst 33342 (blue) staining in tumor sections. Scale bar, 10 μm. (d) Schematic illustration of a mouse bearing a 4T1 tumor treated with CPT for CL imaging. (e) CL images of CPT induction group and control. (f) Quantification of CL intensity for the *in vivo* images in (e). Error bars indicate the s.d. (*n* = 3).

CPT is an antitumor drug with the ability to induce a H_2_O_2_-rich microenvironment in cells.^46^,^47^ To verify that the CPT released in our CLDRS could induce H_2_O_2_ production in tumor cells, we first performed time-dependent measurements of the H_2_O_2_ level in 4T1 breast tumor cells and NIH/3T3 fibroblast cells (the control) *in vitro* with a H_2_O_2_ Assay Kit using 2′,7′-dichlorofluorescin diacetate (DCFH-DA, producing green fluorescence after oxidization by H_2_O_2_). 4T1 tumor cells and 3T3 normal cells were cultured with DCFH-DA for 30 min, and then confocal fluorescence images were recorded over time after adding free CPT, CPT@PEAZO-PCD NPs, CPPO@PEAZO-PCD NPs, and the CLDRSs (Fig. 3a). For 4T1 tumor cells, the relative fluorescence intensity (*F*/*F*_0_, where *F*_0_ indicates the fluorescence intensity at 0 min and *F* is the fluorescence intensity at different time points) for the free CPT group dramatically increased from 1.00 ± 0.11 at 0 min to 9.85 ± 1.13 at 240 min, testifying that CPT in 4T1 cells can cause significant H_2_O_2_ production (Fig. 3b). The PEAZO-PCD NP group showed no influence on the H_2_O_2_ level, while the CPPO@PEAZO-PCD NP group exhibited a rapid decrease because of H_2_O_2_ consumption by CPPO. Thus, we can evaluate the release feasibility of drug CPT in our CLDRS through the *F*/*F*_0_ increase. The negligible *F*/*F*_0_ increase in the CPT@PEAZO-PCD NP group indicated CPT was rarely released into the tumor cells without CL substrate CPPO. Intriguingly, the *F*/*F*_0_ for the CLDRS (CPT + CPPO@PEAZO-PCD NP) group showed an obvious increase from 1.00 ± 0.11 at 0 min to 3.96 ± 0.74 at 240 min (Fig. 3b), demonstrating the effective release of CPT in 4T1 cells by their H_2_O_2_-initiated CL irradiation. Noticeably, this enhanced *F*/*F*_0_ value (∼3.96 fold) was lower than that of the free CPT group (∼11 fold, Fig. 3b) mainly due to the simultaneous consumption of H_2_O_2_ by CPPO in this system. Our results clearly manifested that the CPT release from this CLDRS can be triggered under CL initiation by the pristine H_2_O_2_ level in tumor cells and the released CPT can provide an elevated H_2_O_2_ level for furthering CL enhancement and the resultant self-amplified release. To investigate tumor-specificity of the CLDRS, 3T3 normal cells were used for comparison. We observed no obvious *F*/*F*_0_ increase in normal cells for CPT@PEAZO-PCD NP, CPPO@PEAZO-PCD NP, and CPT + CPPO@PEAZO-PCD NP groups. These results illustrated that the relatively low H_2_O_2_ level in normal cells can hardly produce sufficient CL to isomerize the azobenzene group EAZO and trigger the dissociation of CLDRSs, consistent with CLDRS behavior under similar H_2_O_2_ concentrations *in vitro* (Fig. 2d). It is noted that free CPT can also induce H_2_O_2_ production in normal 3T3 cells (Fig. 2a). Fluorescence of the H_2_O_2_ probe DCFH-DA in free-CPT-treated normal 3T3 cells was obviously lower than that of free-CPT-treated tumor cells at 240 min probably because the initial concentration of H_2_O_2_ was obviously lower than that in the tumor cells. Thus, our CLDRS can promote H_2_O_2_ production only in tumor cells and is inoperative in normal cells, which is beneficial to achieve enhanced CL with excellent tumor specificity.

To further prove the CPT-promoted H_2_O_2_ production *in vivo*, DCFH-DA staining in tumor sections was performed to study the H_2_O_2_ level induced by the released drug CPT. Various NPs were tail-vein injected into mice bearing 4T1 xenograft tumors using PBS as a control. After 12 h, equal amounts of a DCHF-DA working solution were intratumorally injected and then 10 μm cryo-sections of the tumor tissues were prepared and incubated with Hoechst 33342 for nuclei staining. Similar to findings in the cell experiment, the fluorescence intensity of the H_2_O_2_ probe DCFH-DA in the CLDRS group was significantly stronger than that in the control group, indicating that CPT was successfully released from our CLDRS and effectively induced H_2_O_2_ production in tumors (Fig. 3c).

Furthermore, to investigate whether drug CPT-induced H_2_O_2_ production can achieve enhanced CL, we explored the CL intensity and duration in the presence of CPT in tumors. CPT (5 μg mL^–1^) in a 1 : 9 DMSO–PBS solution as a H_2_O_2_ induction group and the 1 : 9 DMSO–PBS solution as control were intratumorally injected into mice bearing 4T1 tumors. After 12 h, equal amounts of a CPT + CPPO + PCD solution were intratumorally injected and both groups were visualized under CL images (Fig. 3d). The CL intensity of the CPT-inducted group was about 10 times more than the control group at 0.5 min (Fig. 3e and f) and obviously stronger than the control group within 30 min, demonstrating that CPT efficiently induced the significantly enhanced CL. Within 30 min, a higher H_2_O_2_ level of the CPT-inducted group consumed more quantitative CPPO than the control group. However, with the same dosage of CL substrate CPPO at the initial status, the CL intensity of the CPT-inducted group was lower than the control after 30 min. Thus, the observed production of high H_2_O_2_ concentrations in tumor cells and tissues by CPT successfully induced its CL enhancement, allowing us to achieve amplified azobenzene EAZO isomerization and construct an amplified drug release system for efficient drug release in tumors.

**Fig. 4:**
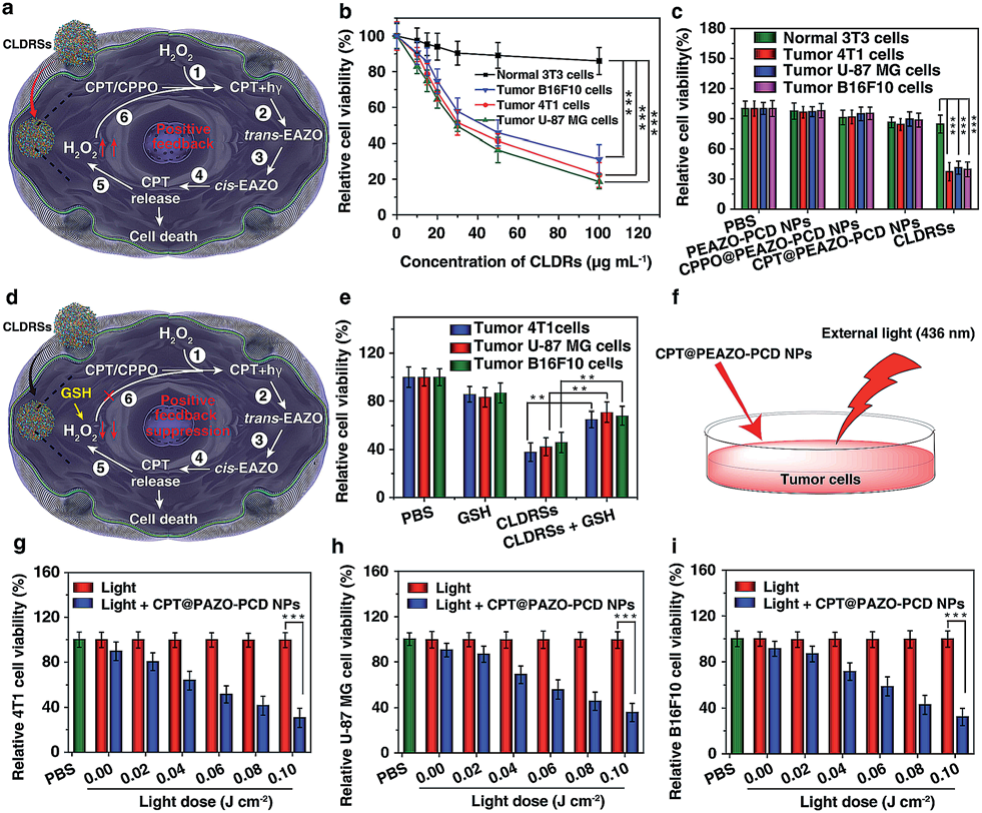
CL-driven photoisomerization for CPT release in *in vitro* antitumor effect. (a) Schematic illustration of positive-feedback-promoted H_2_O_2_ production mechanism and amplified isomerization mechanism of EAZO in cells. (b) Cytotoxicity of normal 3T3 cells as well as tumor 4T1, U-87 MG, and B16F10 cells after 24 h incubation with CLDRSs with different concentrations. ***P < 0.001. (c) Cell viability of normal 3T3 cells as well as tumor 4T1, U-87 MG, and B16F10 cells with various treatments (CLDRSs, 50 μg mL^–1^; equal 9.3 μg mL^–1^ of CPT). ***P < 0.001. (d) Schematic illustration of H_2_O_2_-suppressed mechanism in cells by adding GSH. Cells were incubated with CLDRSs. After 2 hours, the cells were washed twice with PBS and GSH (10 μM) added. (e) Cytotoxicity of CLDRSs (50 μg mL^–1^; equal 9.3 μg mL^–1^ of CPT) treatment with and without GSH. **P < 0.01. (f) Schematic illustration of the chemotherapy efficacy of CLDRSs compared with chemotherapy with external light irradiation. Cell viability of CPT@PEAZO-PCD NPs (equal 9.3 μg mL^–1^ of CPT) with different light dose (436 nm) in tumor (g) 4T1, (h) U-87 MG, and (i) B16F10 cells. ***P < 0.001. Error bars indicate the s.d. (*n* = 3).

Next, the MTT assay was used to determine *in vitro* cytotoxicity of the CLDRSs. The drug carrier PEAZO-PCD NPs at concentrations up to 500 μg mL^–1^ showed no obvious cytotoxicity in normal NIH 3T3 cells and tumor 4T1, U-87 MG, as well as B16F10 cells (Fig. S9†). Just as Fig. 4a illustrated the mechanism of positive-feedback in cells, the CLDRSs indeed exhibited a marked cytotoxicity to tumor 4T1, U-87 MG, and B16F10 cells (Fig. 4b). The half-maximal inhibitory concentration (IC_50_) of CLDRSs was about 35 μg mL^–1^ for tumor 4T1, U-87 MG, and B16F10 cells (Fig. 4b), indicating its good treatment effect. By contrast, the CPT@PEAZO-PCD NPs did not result in a significant decrease in cell viability for 4T1, U-87 MG, or B16F10 cells, indicating a negligible release of CPT in the absence of the CL substrate CPPO (Fig. 4c). Additionally, CPPO@PEAZO-PCD NPs also showed no obvious cytotoxicity for 4T1, U-87 MG, and B16F10 cells (Fig. 4c). These results indicated that EAZO can be isomerized only in CLDRSs for effective drug release. Interestingly, the same experiment performed in normal 3T3 cells with CLDRSs showed no significant decrease in cell viability because low-level-H_2_O_2_-induced weak CL in normal cells was not enough to drive EAZO isomerization for effective drug release (Fig. 4b and c). Additionally, a H_2_O_2_-suppressed experiment was performed. Cells were incubated with CLDRSs. After 2 hours, the cells were treated with glutathione (GSH), an antioxidant and nucleophilic scavenger of H_2_O_2_ (Fig. 4d),^13^ the cell viability returned to a high value for tumor 4T1, U-87 MG, and B16F10 cells (Fig. 4e), indicating that the scavenging of H_2_O_2_ in cells by GSH can weaken the effect of CL-driven azobenzene EAZO isomerization and inhibit drug release from CLDRSs. All these results demonstrated that the CLDRSs possess the potential to achieve tumor-specific EAZO isomerization for tumor-precise therapy *in vivo*, maximizing therapy effects with reduced side effects. The chemotherapy efficacy of CLDRSs compared with chemotherapy with external light irradiation was also evaluated *in vitro* (Fig. 4f). We used CPT@PAZO-PCD NPs containing the same concentration of CPT with CLDRSs to culture tumor 4T1, U-87, and B16F10 cells. After irradiation at 436 nm with different light doses from 0.0 to 0.10 J cm^–2^, chemotherapy of CLDRSs with CL irradiation strategy yielded similar efficacy compared to that with ∼0.08 J cm^–2^ external light irradiation in tumor 4T1, U-87, and B16F10 cells (Fig. 4b, f and g). Additionally, a previous study indicated that PDT with a bioluminescence irradiation strategy yielded similar efficacy compared to that with 0.6–0.8 J cm^–2^ external light irradiation.^24^ Our lower light dose indicated that the CL-driven isomerization strategy may be extended to bioluminescence-driven isomerization.

For an *in vivo* application, we first evaluated the blood retention time of CLDRSs. Due to its lack of imaging signals, a near infrared fluorescence dye, IR825, was encapsulated to form IR825-loaded CLDRS (IR-825 + CPT + CPPO@PEAZO-PCD NPs) for this study (Fig. S10†). Because of the similar size, morphology, and stability of IR825-loaded CLDRS with CLDRSs (CPT + CPPO@PEAZO-PCD NPs) by DLS and TEM observations (Fig. 2b and S11†), IR825-loaded CLDRSs were suitable to modulate our CLDRS for *in vivo* investigations. The blood concentration of IR825-loaded CLDRSs declined to half that level at 8 h after injection (Fig. S12†), showing its long blood retention time. Upon tail-vein injection, the tumor fluorescence signals increased gradually over time and reached a peak value at 5 h after injection, suggesting that IR825-loaded CLDRSs were able to accumulate in tumors (Fig. S13 and S14†). Furthermore, to examine the amount of IR825-loaded CLDRSs in a tumor and other organs, the tumors and major organs of the 4T1 tumor-bearing mice after intravenous injection at 5 h were then taken for *ex vivo* imaging (Fig. S15†). Integrated fluorescence intensity of both the tumor and liver were obviously much stronger than the heart, spleen, lung, and kidney (Fig. S16†). The tumor and liver fluorescence ratio reached up to ∼0.65, demonstrating the high efficiency of tumor accumulation of the CLDRSs *via* an enhanced permeability and retention effect. The high tumor accumulation of the CLDRSs has great potential for improving therapeutic effects.

**Fig. 5:**
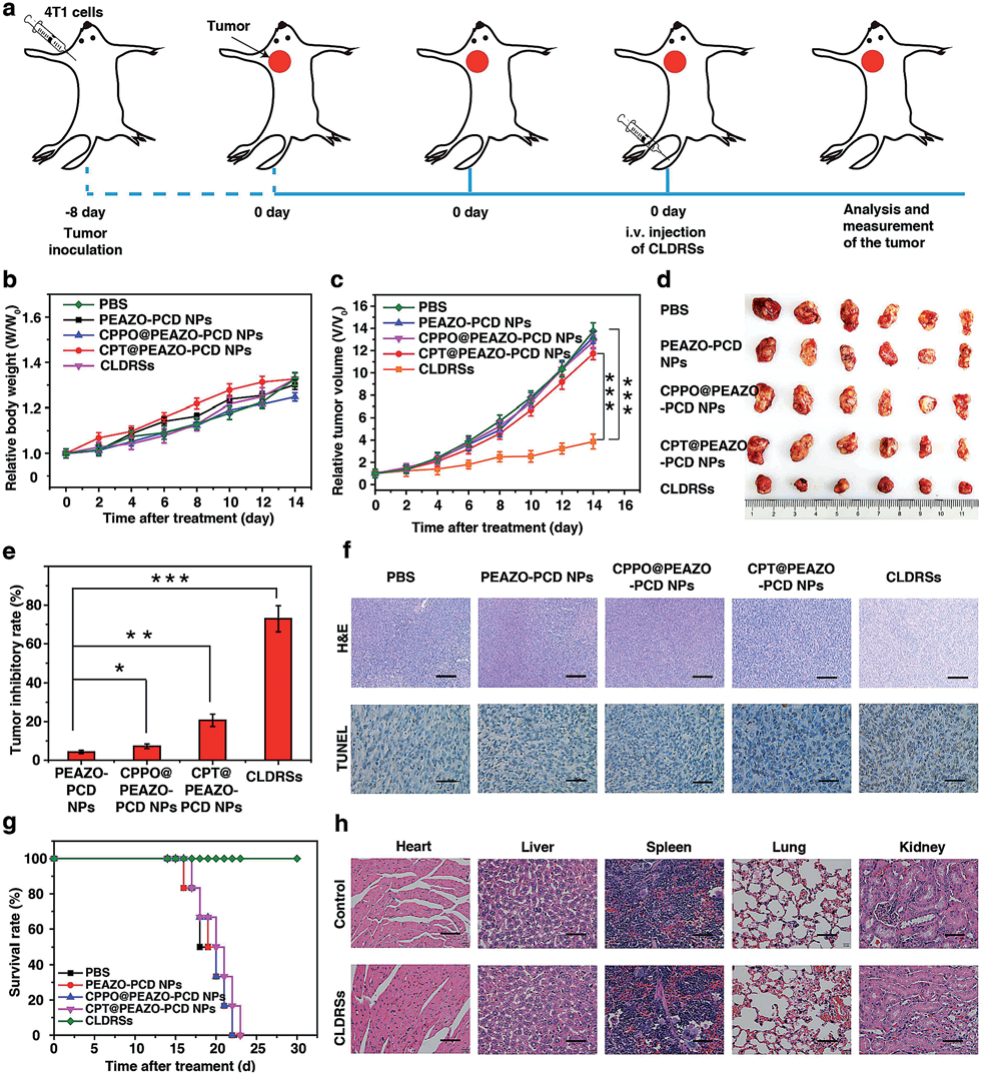
(a) Schematic illustration of CLDRSs therapy to inhibit tumors. (b) Relative body weight and (c) relative tumor volume growth curves. (d) Representative images of tumor tissues after various treatments, respectively. (e) Tumor-inhibitory-rate after various treatments. Error bars indicate standard deviations of 6 separate measurements. Statistical significance: *P < 0.05; **P < 0.01; ***P < 0.001. (f) Tumors were sectioned and stained with H&E and TUNEL. Scale bar, 100 μm. (g) Survival rates of tumor-bearing mice with different treatments within 30 days. (h) H&E stained images of different organs' tissue sections at 30 days after CLDRSs treatment and the healthy mice as control. Scale bar, 30 μm.

To evaluate the therapeutic efficacy *in vivo*, 4T1 tumor-bearing mice were injected *via* tail vein 1 time every 2 days with 200 μL of PEAZO-PCD NPs, CPPO@PEAZO-PCD NPs, CPT@PEAZO-PCD NPs, and CLDRSs at a CPT-equivalent dose of 25 mg kg^–1^ with PBS as a control (Fig. 5a). The tumor volumes and body weights of the tumor-bearing mice were measured every 2 days from 0 to 14 days post injection. After 14 days of treatment, body weights of tumor-bearing mice increased gradually with time (Fig. 5b). Tumor volumes in the mice treated with CLDRSs were much smaller than those in the mice treated with other groups (Fig. 5c and d). Compared with the PBS group, the tumor inhibitory rate (calculated from the tumor volume) in the CLDRS group (73%) is much higher than those in the PEAZO-PCD NP (4%), CPPO@PEAZO-PCD NP (8%), and CPT@PEAZO-PCD NP (21%) groups (Fig. 5e). These results demonstrated that CL effectively drove azobenzene group EAZO photoisomerization to implement drug-controlled release for excellent therapeutic efficacy against tumors.

To further evaluate the antitumor efficacy of treatments with various NPs, immunohistochemical analysis was performed. In the CLDRS-treated groups, tumor cells displayed nuclear shrinkage and fragmentation and, most importantly, a large necrotic area (Fig. 5f). Conversely, obvious malignant necrosis was not observed in the other groups. Similar results were obtained by TdT-mediated dUTP Nick-End Labelling (TUNEL) analysis (Fig. 5f). Therefore, both H&E and TUNEL analysis further confirmed the excellent antitumor efficacy of the CLDRS *in vivo*.

Potential *in vivo* side effects of the CLDRSs were further evaluated. After 14 days of treatment, body weights of tumor-bearing mice increased gradually with time, illustrating almost negligible side effects of CLDRSs for tumor therapy (Fig. 5b). After 30 days of treatment, the survival rate of mice treated with the CLDRSs remained at 100%, which was significantly improved compared with the 0% survival rate in other groups (Fig. 5g). Next, long-term damage to major organs was also assessed. Due to the self-repairing damage capability of organs, at 30 days after treatment, the major organ slices stained with H&E exhibited no noticeable signs of organ damage (Fig. 5h). These results confirmed the high selectivity for tumors and minimal off-target damage from the CLDRSs used for *in vivo* therapy. Such no obvious side-effects also proves that enhanced CL effectively achieved effective azobenzene photoisomerization at precise target sites.

## Conclusions

In summary, we have demonstrated the concept of target-specific self-luminescence-driven photoisomerization *in vivo* by developing a target-specific initiated and *in situ*-enhanced CL strategy that solves restrictions of lacking a target-specific high-intensity self-luminescence. Furthermore, we first demonstrated the applicability of our strategy to construct target-specific self-luminescence-controlled drug release systems for tumor chemotherapy. Our target-specific enhanced mechanism not only conveniently enhanced the CL to provide sufficient energy, but also tactfully ensured target-specific enhancement. As we know, target-specific photoisomerization with external photoexcitation completely relies on a selective illumination region with artificial consciousness. It is difficult to ensure an accurate position in practical applications because most target tissues (*e.g.*, tumor) have small areas and irregular shapes. In comparison, our target-specific initiated and enhanced CL had abilities of self-selected target tissue regions through a high H_2_O_2_ level in diseased tissues.

Although we only demonstrated one example of a photoisomerization-related bioapplication, namely CL-controlled drug chemotherapy, our work of CL-driven isomerization provides a common guideline to design various target-specific depth-independent photoisomerization in bioapplications. In this CL-driven isomerization system, its CL wavelength was determined by the fluorescence of the fluorophore itself and the light wavelength of *trans*–*cis* photoisomerization of EAZO was within a fairly large wavelength range (<570 nm).^42^,^43^ Thus, we can easily select applicable fluorophores as needed to replace fluorophore CPT. Additionally, for a CL enhancer, some non-drug enhancers (*e.g.*, d-amino acid oxidase^48^ or vitamin C and its derivatives^49^) also can be linked with a disease-microenvironment-specific response group to induce H_2_O_2_ production only in target tissue to achieve target-specific enhanced CL for other non-treatment applications. Considering that many diseases, such as inflammation, neurodegenerative diseases, and chronic obstructive pulmonary diseases,^13^ are closely associated with a high H_2_O_2_ level, our CL-driven isomerization strategy may therefore be applied to these diseases.

Moreover, we can also use other self-luminescence to isomerize this azobenzene due to their similar light energy levels, thus providing new advantages for photoisomerization-related studies. For instance, we can use genetic engineering to accomplish target-specific bioluminescence for this azobenzene isomerization *in vivo*. Furthermore, our proposed target-specific initiated an *in situ*-enhanced self-luminescence method by a positive feedback mechanism which provided a robust and versatile approach to implement target-specific enhanced self-luminescence, which will greatly broaden the application of self-luminescence. In future work, our self-luminescence-driven isomerization strategy may be generalized to explore photo-switchable biomolecules for a variety of *in vivo* applications, such as those used in artificial intelligence, optogenetics, and even the photo-controlling every aspect of a cell's inner workings, in a depth-independent manner with high target specificity, thus prospectively overcoming the Achilles' heel of photoisomerization.

## Ethical statement

All animal experiments of this study were performed at the Experimental Animal Center of Simcere Pharmaceutical Group in full compliance with the guidelines approved by Jiangsu Administration of Experimental Animals. Approximately 3 × 10^6^ 4T1 cells in 200 μL of PBS were inoculated in 5 week-old 4T1 female Balb/c nude mice by subcutaneous injection into their right limb armpits.

All tumor bearing nude mice were purchased from Jiangsu KeyGEN BioTECH Corp., Ltd. and used according to the guidelines of the Laboratory Animal Center of Jiangsu KeyGEN BioTECH Corp., Ltd.

## Author contributions

Y. T. and Q. F. conceived, supervised, and designed the study; Y. T. and X. L. performed experiments. Other people analyzed data and wrote the manuscript.

## Conflicts of interest

There are no conflicts to declare.

## Supplementary Material

Supplementary information
