# Supplementary material for: Chemiluminescence-initiated and in situ-enhanced photoisomerization for tissue-depth-independent photo-controlled drug release
Source: Chem Sci. 2018 Nov 10;10(5):1401–9. doi: 10.1039/c8sc04012e (PMC6354828; doi:10.1039/c8sc04012e)
Supplement: Supplementary file 1 [file SC-010-C8SC04012E-s001.pdf]

Electronic Supplementary Material (ESI) for Chemical Science.
This journal is © The Royal Society of Chemistry 2018

Chemiluminescence-initiated and in-situ-enhanced photoisomerization for tissue-depth-independent photo-controlled drug release

Yufu Tang, Xiaomei Lu, Chao Yin, Hui Zhao, Wenbo Hu, Xiaoming Hu, Yuanyuan Li, Zhen Yang, Feng Lu, Quli Fan, and Wei Huang*

Materials

All commercially available reagents and solvents were obtained from Sigma-Aldrich (Shanghai, China) and used without further purification, unless noted otherwise. Camptothecin (CPT) and Hydrogen peroxide (H_2O_2) were purchased from Aldrich (Shanghai, China). Bis(2-carbopentyloxy-3,5,6-trichlorophenyl) oxalate (CPPO) and 3A-Amino-3A-deoxy-(2AS,3AS)-β-cyclodextrin Hydrate (NH_2-β-CD) were purchased from TCI (Shanghai, China). Reactive oxygen species (ROS) kit (DCFH-DA) was purchased from Thermo Fisher (Shanghai, China).

Measurements

^{1}H and ^{13}C nuclear magnetic resonance spectroscopy (^{1}H NMR, 400 MHz; and ^{13}C NMR, 400 MHz) spectra were recorded using a Bruker Ultra Shield Plus 400 MHz spectrometer with deuterium chloroform ($CDCl_3$) and DMSO-d6 as a solvent. (^{1}H NMR, 400 MHz; and ^{13}C NMR, 400 MHz) spectra were used tetramethylsilane (TMS) as the internal standard, and the following abbreviations represent the multiplicities: s = singlet, d = doublet, t = triplet, q = quartet, m = multiplet. Mass spectra were obtained on a matrix-assisted laser desorption/ionization time of flight mass spectrometry MS (MALDI-TOF, Bruker AutoFlex III system). The ultraviolet-visible (UV-vis) absorption, fluorescence and chemiluminiscence spectra were measured by a Shimadzu UV-vis spectrophotometer (UV-3600) and fluorescence spectrophotometer with a photomultiplier tube detector, respectively. The molecular weights (Mn) and polydispersities (PDI) of the polymers were determined by gel permeation chromatography (GPC) analysis on a Shim-pack GPC-80X column with water as the

eluent at a flow rate of 1.0 mL min^{-1}. Dynamic light scattering (DLS) was performed on a particle size analyzer (NanoBrook 90Plus, Brookhaven Instruments Corporation). Transmission electron microscopy (TEM) images were performed on a HT7700 transmission electron microscope operating at 100 kV. The methyl thiazolyl tetrazolium (MTT) assay was performed by a PowerWave XS/XS2 microplate spectrophotometer (BioTek, Winooski, VT). Images were acquired by IVIS living imaging system.

Statistical analysis

Statistical analyses of the data were performed with the SPSS 12.0 software. Measurement data are expressed as the mean ± SD. For cell viability assays, a Student's t-test was applied to identify significant differences. Multiple group comparison was conducted by one-way analysis of variance (ANOVA). A value of $p < 0.05$ was considered statistically significant.

Methods

Synthetic route to the compound PEAZO and PCD.[1, 2]

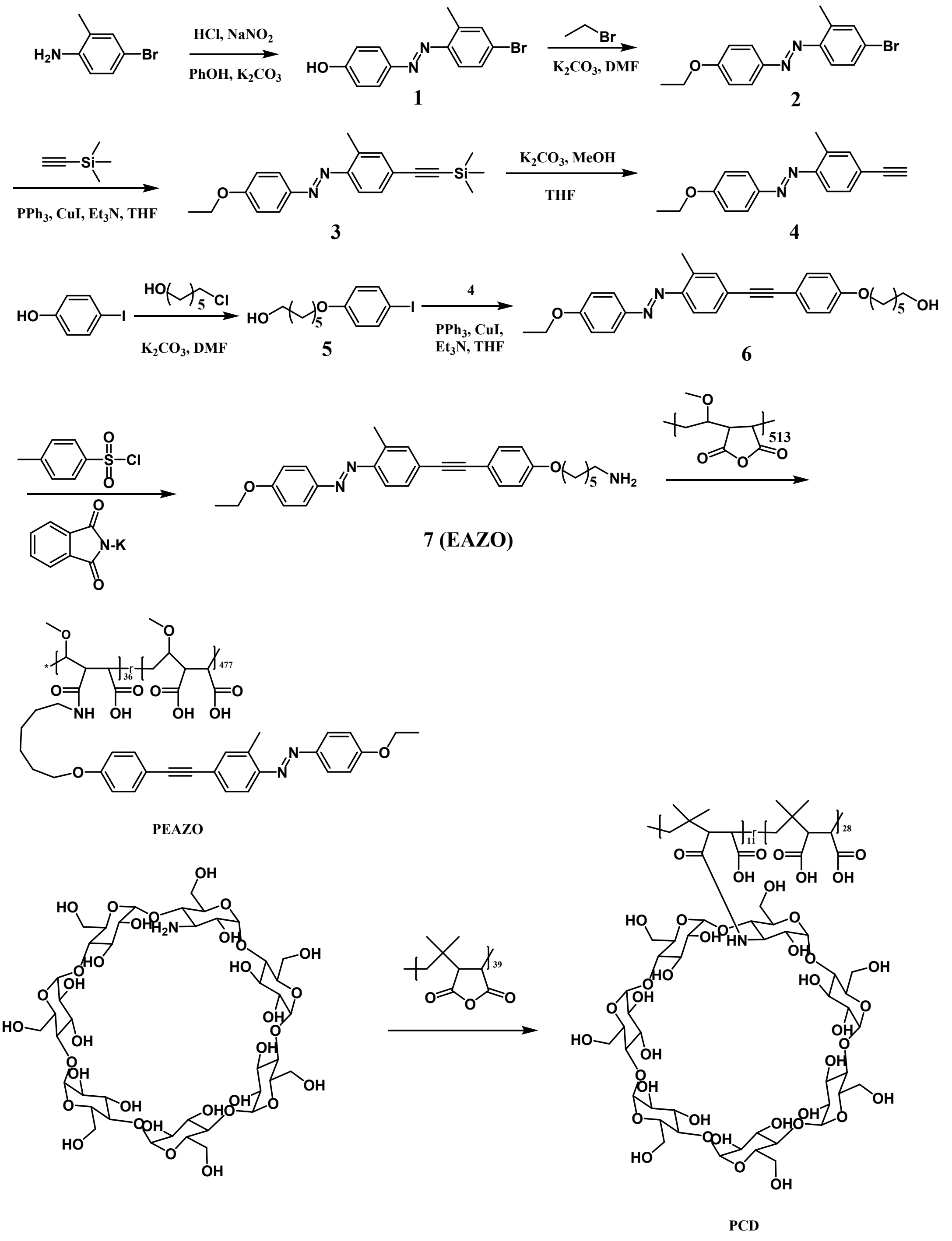

Synthesis of 1.

Monomer 1 was synthesized according to previous report.[3, 4] 4-Bromo-2-methylaniline

(2.23 g, 12 mmol) was dissolved in 3.7 mL concentrated hydrochloric acid and 30 mL water in ice-bath. With vigorous stirring, a solution of sodium nitrite (0.827 g, 12 mmol) was dropwise added into the cold solution. After 30 min, a mixture of phenol (1.128 g, 12 mmol) and potassium carbonate (1.659 g, 12 mmol) in 6 mL water was added dropwise to the resulting diazonium solution. After 3 h, the solution was adjusted to the pH = 7.0. The precipitate was collected and purified via silica gel column chromatography (hexane/ethyl acetate = 5/1) (Yield: 86%). ^{1}H-NMR (400 MHz, $CDCl_3$, d, ppm): 2.66 (s, 3H), 7.02 (d, 2H), 7.58 (d, 1H), 7.73–7.77 (m, 4H). MALDI-TOF-MS: m/z calcd for $[M + Na]^+$ $C_{13}H_{10}BrN_2NaO$, 313.13, found 313.56.

Synthesis of 2.

Monomer 2 was synthesized according to previous report.[4] Monomer 1 (1.746 g, 6 mmol), potassium carbonate (0.8295 g, 6 mmol) and iodoethane (0.936 g, 6 mmol) was dissolved in DMF (15 mL). The solution was stirred for 24 h at 60 ^{0}C and cooled to room temperature. Then the reaction mixture was poured into 500 mL water and filtered to get the precipitate, which was then purified via silica gel column chromatography (hexane/ethyl acetate = 3/1) (Yield: 79%). ^{1}H NMR (400 MHz, $CDCl_3$) δ 7.92 – 7.86 (m, 2H), 7.49 (dd, J = 8.2, 5.2 Hz, 2H), 7.37 (dd, J = 8.6, 2.1 Hz, 1H), 7.03 – 6.94 (m, 2H), 4.12 (q, J = 7.0 Hz, 2H), 2.68 (s, 3H), 1.46 (t, J = 7.0 Hz, 3H). MALDI-TOF-MS: m/z calcd for $[M + H]^+$ $C_{15}H_{16}BrN_2O$, 320.20, found 319.67.

Synthesis of 3.

Monomer 3 was synthesized according to previous report.[4] Compound 2 (2.23 g, 7.0 mmol), trimethylsilylacetylene (1.3 g, 3.07 mmol), $PdCl_2(PPh_3)_2$ (0.233 g, 0.33 mmol),

CuI (0.233 g, 1.2 mmol), and PPh3 (0.43 g, 1.67 mmol) were dissolved in triethylamine (10 mL) and THF (10 mL). The mixture was stirred at 65 °C for 24 h under a nitrogen atmosphere and cooled to room temperature. The reaction mixture was extracted with dichloromethane. Then the dichloromethane layer was collected and dried with anhydrous magnesium sulfate, the solvent was removed by evaporation. The product was purified by silica gel column chromatogrqaphy (ethyl acetate/hexane = 2:1) (yield = 85%). ^{1}H NMR (400 MHz, CDCl3) δ 7.93 – 7.87 (m, 2H), 7.56 (d, J = 8.3 Hz, 1H), 7.46 – 7.32 (m, 2H), 7.04 – 6.96 (m, 2H), 4.12 (q, J = 7.0 Hz, 2H), 2.66 (s, 3H), 1.46 (t, J = 7.0 Hz, 3H), 0.30 – 0.24 (m, 9H). ^{13}C NMR (101 MHz, $CDCl_3$) δ 161.58 (s), 150.32 (s), 137.51 (s), 134.72 (s), 130.12 (s), 124.86 (d, *J* = 18.6 Hz), 115.36 (s), 114.71 (s), 105.07 (s), 96.10 (s), 77.33 (s), 77.02 (s), 76.70 (s), 63.85 (s), 17.32 (s), 14.77 (s), -0.01 (d, *J* = 3.5 Hz). MALDI-TOF-MS: m/z calcd for $[M + H]^+$ $C_{20}H_{25}N_2OSi$, 336.51, found 336.84.

Synthesis of 4.

Monomer 4 was synthesized according to previous report.[4] Monomer **3** (2.0 g, 6 mmol) and potassium carbonate (0.033 g, 0.239 mmol) were dissolved in a mixture of THF (6 mL) and methanol (65 mL). The solution was stirred for 4 h at room temperature. The mixture was extracted with ethyl acetate. After the organic layer was dried with anhydrous magnesium sulfate, the solvent was removed by evaporation. The product was purified by silica gel column chromatogrqaphy (ethyl acetate/chloroform = 1:1) (yield = 90%). ^{1}H NMR (400 MHz, $CDCl_3$) δ 7.94 – 7.88 (m, 2H), 7.58 (d, *J* = 8.3 Hz, 1H), 7.48 – 7.35 (m, 2H), 7.03 – 6.97 (m, 2H), 4.13 (q, *J* = 7.0 Hz, 2H), 3.18 (s, 1H),

2.67 (s, 3H), 1.46 (t, *J* = 7.0 Hz, 3H). ^{13}C NMR (101 MHz, $CDCl_3$) δ 161.65 (s), 150.62 (s), 147.35 (s), 137.52 (s), 134.87 (s), 130.30 (s), 125.00 (s), 123.67 (s), 115.50 (s), 114.72 (s), 83.66 (s), 78.66 (s), 77.34 (s), 77.02 (s), 76.71 (s), 63.86 (s), 17.35 (s), 14.77 (s), 0.01 (s). MALDI-TOF-MS: m/z calcd for $[M + H]^+$ $C_{17}H_{18}N_2O$, 266.33, found 265.59.

Synthesis of 5.

A mixture of 4-iodophenol (2.2 g, 10 mmol), 6-chlorohexan-1-ol (1.36 g, 10 mmol) and potassium carbonate (1.5 g, 11 mmol) was dissolved in DMF (30 mL). The reaction mixture was refluxed with stirring for 3 h and cooled to room temperature. Water was added to the mixture and the precipitate was collected. The crude product was purified via silica gel column chromatography (hexane–ethyl acetate = 2/1) (Yield: 76%). ^{1}H NMR (400 MHz, $CDCl_3$) δ 7.58 – 7.47 (m, 2H), 6.70 – 6.60 (m, 2H), 3.90 (t, J = 6.5 Hz, 2H), 3.65 (dd, J = 8.8, 4.3 Hz, 2H), 1.77 (dq, J = 13.2, 6.5 Hz, 2H), 1.63 – 1.52 (m, 2H), 1.50 – 1.35 (m, 4H). ^{13}C NMR (101 MHz, $CDCl_3$) δ 158.95 (s), 138.17 (s), 116.93 (s), 82.50 (s), 77.40 (s), 77.08 (s), 76.76 (s), 67.96 (s), 62.85 (s), 32.61 (s), 29.12 (s), 25.85 (s), 25.53 (s), 24.96 (s), 1.05 (s), 0.04 (s). MALDI-TOF-MS: m/z calcd for $[M + H]^+$ $C_{12}H_{18}IO_2$, 321.17, found 320.49.

Synthesis of 6.

Monomer 6 was synthesized according to previous report.[4] Compound **5** (2.9 g, 9.1 mmol), compound **4** (2.0 g, 7.6 mmol), $PdCl_2(PPh_3)_2$ (0.27 g, 0.38 mmol), CuI (0.27 g, 0.98 mmol), and PPh_3 (0.37 g, 1.9 mmol) were dissolved in trimethylamine (30 mL) and THF (30 mL). The mixture was stirred at 60 °C for 8 h under a nitrogen atmosphere.

The resulting solution was cooled to room temperature and extracted with ethyl acetate. After the organic layer was dried with anhydrous magnesium sulfate, the solvent was removed by evaporation. The product was purified by column chromatogrqaphy (silica gel, ethyl acetate: hexane = 1:1 as eluent) to yield 2.0 g (58%) of orange powder. ^{1}H NMR ($CDCl_3$, δ, ppm): 1.43-1.85 (m, 8H), 1.93 (s, 3H), 2.66 (s, 3H), 4.02-4.18 (m, 4H), 5.54 (s, 1H), 6.09 (s, 1H), 6.85 (d, J) 8.8 Hz, 2H). MALDI-TOF-MS: m/z calcd for $[M + H]^+$ $C_{29}H_{33}N_2O_3$, 457.59, found 456.63.

Synthesis of 7 (EAZO).

Monomer 7 was synthesized according to previous report. 6 (9.0 g, 20 mmol), tosyl chloride (11.40 g, 60 mmol) and pyridine (20 mL) were dissolved in dichloromethane (250 mL). The resulting solution was stirred at 20 °C for 16 h. The reaction mixture was diluted with water (200 mL). The organic layer was washed with equivalent volumes of water several times. The organic layer was washed with brine twice and dried over magnesium sulfate, the solvent was evaporated under reduced pressure, and the crude product was used the next step. The crude product was used the next step. The crude product (5.85 g, 10 mmol) and potassium phthalimide (2.09 g, 11.28 mmol) were dissolved in dimethylformamide (250 mL) and stirred at 80 °C for 16 h. Afterward, the reaction mixture was allowed to cool to room temperature and was diluted with ethyl acetate (250 mL). The organic layer was washed with equivalent volumes of water several times; the combined aqueous phases were extracted twice with ethyl acetate (150 mL) each. The organic layer was washed with brine twice and dried over magnesium sulfate, the solvent was evaporated under reduced pressure, and

the crude product dissolved in ethanol (20 mL) was cooled to 0 °C, and hydrazine hydrate (1 mL) was added slowly. After stirring for 15 min at 0 °C the reaction was refluxed for 3 h. The reaction was allowed to cool to room temperature, diluted with 4 M HCl (200 mL), and filtered, and the filtrate was washed with dichloromethane (100 mL) before being basified to pH 14 with concentrated NaOH solution. The water phase was extracted five times with dichloromethane (100 mL), the extracts were combined and dried over Na_2SO_4, and the solvent was evaporated to give the desired compound in 61% yield. ^1H NMR (400 MHz, DMSO) δ 7.88 (d, J = 8.9 Hz, 2H), 7.56 (d, J = 8.0 Hz, 2H), 7.48 (d, J = 8.8 Hz, 2H), 7.42 (d, J = 8.6 Hz, 1H), 7.11 (d, J = 9.0 Hz, 2H), 7.00 – 6.94 (m, 2H), 4.13 (q, J = 7.0 Hz, 3H), 3.99 (t, J = 6.6 Hz, 3H), 2.64 (s, 3H), 1.73 – 1.68 (m, 2H), 1.39 – 1.31 (m, 9H). MALDI-TOF-MS: m/z calcd for $[M + H]^+$ $C_{29}H_{34}N_3O_2$, 456.60, found 455.87.

Synthesis of PEAZO.

Polymer PEAZO was synthesized according to previous report.[2] Poly(methyl vinyl ether-*alt*-maleic anhydride) (800 mg, Mn = 80 kDa), 7 (100 mg) and DIPEA (1 mL) were dissolved in 200 mL of dry THF. The mixture was stirred for 16 h at 50 °C. After THF evaporation, the material was resuspended in water with small excess of NaOH with respect to the carboxylic groups on the polymer backbone. After evaporation of water and DIPEA, the remaining residue was dissolved in water. Then the solution was dialyzed (dialysis membrane MWCO 3500) against DI water for 3 day. After dialysis, the result solution was filtered through a 0.45 μm filter and then lyophilized at room temperature for 3 days to obtain a yellow powder. The conjugation ratios of PEAZO

within each the backbone polymer chain were about 36 EAZO molecules. (GPC: Mn = 95 kDa, PDI = 1.56).

Synthesis of PCD.

Polymer PCD was synthesized according to previous report.[2] Poly(isobutylene-alt-maleic anhydride) (500 mg, Mn = 6 kDa), 3A-Amino-3A-deoxy-(2AS,3AS)-β-cyclodextrin Hydrate (NH_2-β-CD, 48.7 mg) and DIPEA (1 mL) were dissolved in 200 mL of dry N,N-Dimethylformamide. The mixture was stirred for 16 h at 50 °C. The material was resuspended in water with small excess of NaOH with respect to the carboxylic groups on the polymer backbone. After evaporation of water and DIPEA, the remaining residue was dissolved in water. Then the solution was dialyzed (dialysis membrane MWCO 3500) against DI water for 3 day. After dialysis, the result solution was filtered through a 0.45 μm filter and then lyophilized at room temperature for 3 days to obtain a powder. The conjugation ratios of PCD within each the backbone polymer chain were about 11 β-CD-NH_2 molecules. (GPC: Mn = 18 kDa, PDI = 1.49).

The emission-reabsorption studies

The emission-reabsorption process using an experimental setup as previously reported.[4] CPT, CPPO, PCD and H2O2 (2 mM) in beaker to produce blue CL, and the generated CL emission to irradiate a PBS solution of the PEAZO-PCD NPs in a highly optically transparent cell. The no CL of CPT as control through equal PBS instead of H_2O_2 in a beaker, everything else is absolutely identical. Highly optically transparent cell was continuously irradiated three times by blue CL. Each time the beaker is filled with fresh CPT, CPPO, PCD and H_2O_2 (2 mM).

Preparation of PEAZO-PCD NPs.

PEAZO (36 mg) and PCD (12 mg) (molar ratio of EAZO/β-CD-NH_2 = 1:2) was dissolved in mlli-Q water (8 mL), and stirred for 24 h at room temperature in darkness. The supernatant (PEAZO-PCD NPs) was filtered through a 0.22 μm filter. Then, the homogeneous PEAZO-PCD NPs solution was lyophilized.

Drug loading on nanoparticles. PCD (36 mg) was dissolved in mlli-Q water (8 mL), and then added PEAZO (12 mg). The resulting solution was slowly added into 2 mL DMSO solution containing CPT (5 mg mL^{-1}) and CPPO (5 mg mL^{-1}), and stirred for 24 h at room temperature in darkness. The supernatant was filtered through a 0.22 μm filter to remove the excess CPT and CPPO. Then, the resulting filtrate was diluted and stored in fridge at 4 ^{o}C for further use.

CPT releasing in vitro. Methods were demonstrated as previous reports.[5, 6] The release profiles were studied by monitoring the increase of CPT concentration in culture solution over time by high-performance liquid chromatography (HPLC) using a UV detector. In a typical procedure, 3 mL prepared CLDRSs or CPT@PEAZO-PCD NPs solution were transferred into a presoaked dialysis cassette (Slide-A-Lyzer, MWCO 10 kDa, Pierce Biotechnology, Rockford IL). The cassette was allowed to stir in a beaker containing 500 mL PBS (pH 7.4 at 37 °C) in the absence or presence of different concentrations H_2O_2 (0 μM, 10^{-4} mM, 0.05 mM, 0.5 mM and 1 mM). At designated time intervals, 50 mL of medium was taken out from the culture solution and concentrated by vacuum rotary. The concentration of the released drug was analyzed by HPLC based on the standard curve calibrated with a drug solution with a known

concentration. The release was evaluated in triplicate.

Cell Cultures. 3T3, 4T1, U-87 MG and B16F10 cells were cultured in Dulbecco's modified Eagle's medium (DMEM) containing 10% fetal bovine serum (FBS) and 1% penicillin/streptomycin. Cells grew as a monolayer and were detached upon confluence using trypsin (0.5% w/v in PBS). The cells were harvested from the cell culture medium by incubating in a trypsin solution for 5 min. The cells were centrifuged, and the supernatant was discarded. 3 mL portion of serum-supplemented DMEM was added to neutralize any residual trypsin. The cells were resuspended in serum-supplemented DMEM at a concentration of 1×10^4 cells/mL. Cells were cultured at 37 °C and 5% CO_2.

Cytotoxicity assay.

The cell viability of PEAZO-PCD NPs was first evaluated by employing the MTT viability assay on the normal NIH 3T3 cells and tumor 4T1, U-87 MG as well as B16F10 cells incubated with PEAZO-PCD NPs at 0, 50, 100, 200, 400 and 500 μg/mL for 24 h. In vitro cytotoxicity study of CLDRSs was then quantitatively evaluated by employing the MTT viability assay on 4T1 tumor cells. Cells were trypsinized and plated onto 96-well plates (1×10^4 per well), respectively. Then, 4T1 cells were exposed to a series of concentrations of CLDRSs at 0, 10, 15, 20, 30 and 100 μg/mL for 24 h. Then, cytotoxicity was quantitatively evaluated by employing the MTT viability assay on 4T1 tumor cells.

Cytotoxicity of CPT@PEAZO-PCD NPs with external 436 nm Light Irradiation in tumor 4T1, U87 and B16F10 cells. For cytotoxicity studies, 4T1, U87 and B16F10

cells were seeded into 96-well plates at a density of 10,000 cells per well and cultured for 24 hours. Cells were treated with CPT@PEAZO-PCD NPs (equal 9.3 μg mL^{-1} of CPT). Then, these cells were incubated at 37 °C under 5% CO2. After 3 hours, the cells were washed twice with phosphate buffered saline (PBS). The phototoxicity of 4T1, U87 and B16F10 cells were irradiated by 436 nm LED with different light doses from 0 to 0.1 J cm^{-2}. After incubation for 24 hours, cytotoxicity was determined using the MTT assay.

CPT-induced H_2O_2 production. 1 × 10^4 4T1 cells/well was cultured with fresh DMEM medium with 10% FBS, then ROS kit (10 μM) was added into the medium. After 30 min, CPT@PEAZO-PCD NPs (15 μg mL^{-1}, equivalent to a local CPT concentration of 5 μg/mL), CPPO@PEAZO-PCD NPs (15 μg mL-1) and CLDRSs (26.9 μg mL^{-1}, equivalent to a local CPT concentration of 5 μg/mL) was added into medium, respectively. Free CPT (1 mg/mL in DMSO) was added into another medium (the final concentration of CPT, 5 μg/mL). After 1 h of incubation, then cells were imaged at different time points: 0 min, 90 min, 120 min, 150 min, 180 min, 210 min and 240 min with confocal laser scanning microscopy (CLSM) (Leica TCS SP5, Germany) for insight on dichlorofluorescein (DCF) decomposed from DCFH-DA (λ_{ex} = 488 nm, λ_{em} = 490 nm–550 nm, respectively). The 3T3 normal cell was performed the same method for comparison. The fluorescence intensity was further analyzed with Image J software.

4T1 Tumor Animal models.

In vivo blood elimination kinetics. Methods were demonstrated as previous reports.

The jugular vein of Female BALB/c nude mice bearing 4T1 tumors (~90 mm^3) was cannulated and a catheter was implanted for intravenous injection and blood collection (Harland, Indianapolis, IN, USA). IR825-loaded CLDRSs were injected through the catheter at a dose of 5 mg kg^{-1} body weight (n = 3 for each group). Whole blood samples (~100 μL) were collected via jugular vein catheter before dosing and at predetermined time points post injection and used to detect IR825 concentration by the measurement of absorbance at 800 nm on a Shimadzu UV-vis spectrophotometer (UV-3600). The values were plotted versus time after the subtraction of blood background.

Fluorescence imaging. Female BALB/c nude mice bearing 4T1 tumors developed established tumor (6–10 mm in diameter), they were subjected to in vivo fluorescence imaging by injecting of IR825-loaded CLDRSs (1 mg mL^{-1},100 μL) via tail vein. At different time points post injection of IR825-loaded CLDRSs, mice were scanned using an IVIS living imaging system. An excitation bandpass filter at 780 nm and an emission at 800 nm under anaesthesia. After in vivo imaging, animals were killed. Tumor and major organs were excised and imaged with the IVIS living imaging system after tail vein injection of IR825-loaded CLDRSs for 5 h.

***In vivo* therapeutic studies.** Female BALB/c nude mice bearing 4T1 tumors (~90 mm^3) were administered intravenously with 200 μL of PBS,PEAZO-PCD NPs, CPPO@PEAZO-PCD NPs, CPT@PEAZO-PCD NPs and CLDRSs (25 mg/kg on a CPT base) for 1 time every 2 days. Tumor progression and body weight of mice were monitored on alternative days. The tumor progression was monitored individually by measuring tumor size with a digital vernier caliper. The individual tumor volume (V)

was calculated according to the following formula: $V = (a \times b^2) / 2$, where length (a) is the longer dimension of tumor tissues and width (b) is the shorter dimension perpendicular to length.

***Ex Vivo* Histological Staining.** At day 14 post-drug administration, the mice were sacrificed for organ collection. The excised organs were immersed in OTC and transferred for cryosection to obtain 10 μm thick slices for histological analysis. The hematoxylin-eosin (H&E) and the terminal deoxynucleotidyl transferase dUTP nick end labeling (TUNEL) staining assay were performed according to standard protocol. Observation was performed under fluorescent microscope (Olympus inverted microscope IX-71).

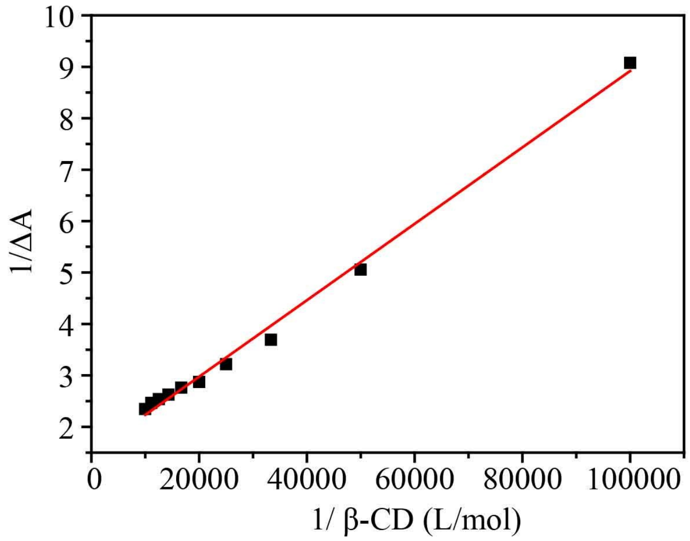

Figure S1. Double reciprocal plots of the supramolecular inclusion complexes in water:[7] The complex constant (K_c) calculated of β-CD-NH_2 cyclodextrin with EAZO precursor (E)-1-(4-bromo-2-methylphenyl)-2-(4-ethoxyphenyl)diazene (R^2 = 0.99471). The Benesi-Hildebrand equation is expressed as $1/\Delta A = 1/\alpha + 1/\alpha K_c$[β-CD-

$NH_2]^n$, where ΔA is the change of UV absorbance of EAZO precursor in presence of β-CD-NH_2, α is a constant, [β-CD-NH_2] is the initial concentration of β-CD-NH_2. K_c is the constant for the formation of n : 1 (Host : Guest) inclusion complex, which could be calculated from a plot of 1/ΔA versus EAZO/[β-CD-$NH_2]^n$.

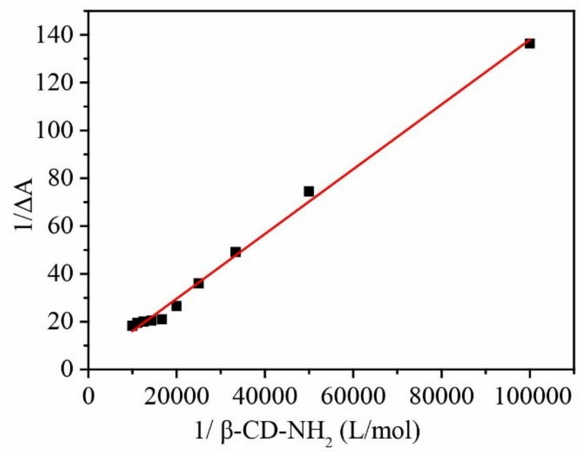

Figure S2. Double reciprocal plots of the supramolecular inclusion complexes in water:[7] The complex constant (K_c) calculated of β-CD-NH_2 cyclodextrin and CPPO (R^2 = 0.99471). The Benesi-Hildebrand equation is expressed as 1/ΔA = 1/α+ 1/αK_c[β-CD-$NH_2]^n$, where ΔA is the change of UV absorbance of CPPO in presence of β-CD-NH_2, α is a constant, [β-CD-NH_2] is the initial concentration of β-CD-NH_2. K_c is the constant for the formation of n : 1 (Host : Guest) inclusion complex, which could be calculated from a plot of 1/ΔA versus CPPO/[β-CD-$NH_2]^n$.

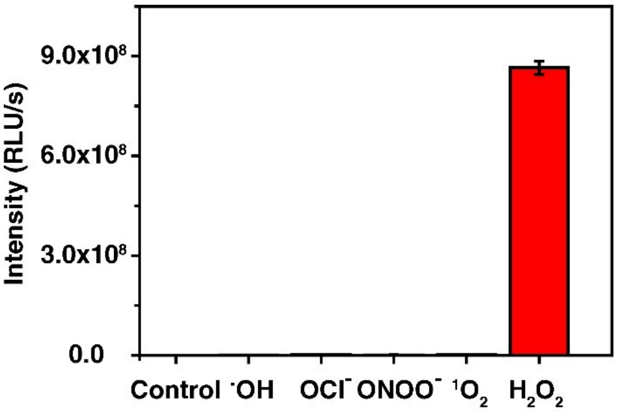

Figure S3. Specificity test of the chemiluminescence signal of CPT (5 μg mL^{-1}, 1 mL), CPPO (5 μg mL^{-1}, 1 mL) and PCD (10 μg mL^{-1}, 1 mL) in the presence of different kinds of reactive oxygen species in a 1:9 DMF-PBS solution.

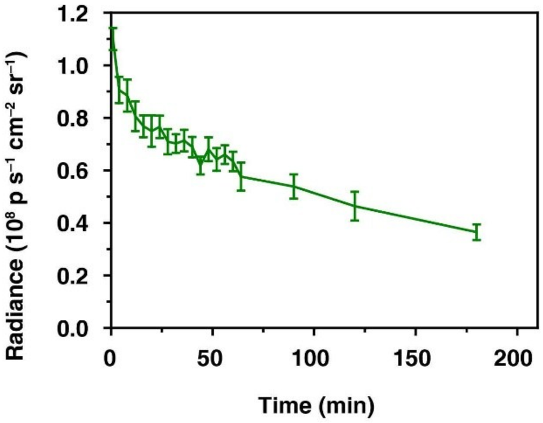

Figure S4. CL signal stability of CPT (25 μg mL^{-1}, 1 mL) and CPPO (25 μg mL^{-1}, 1 mL) with PCD (50 μg mL^{-1}, 1 mL) in a 1:9 DMF-PBS solution after adding H_2O_2 (1 mM). Error bars, Standard deviation (s.d.) (n = 3 replicates).

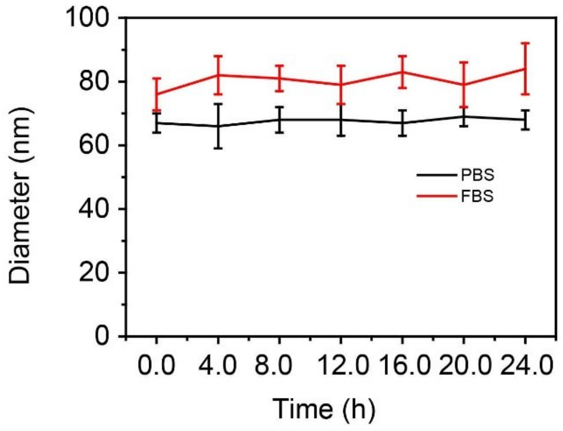

Figure S5. The change in DLS of CPT@PEAZO-PCD NPs after storage under dark over time in PBS.

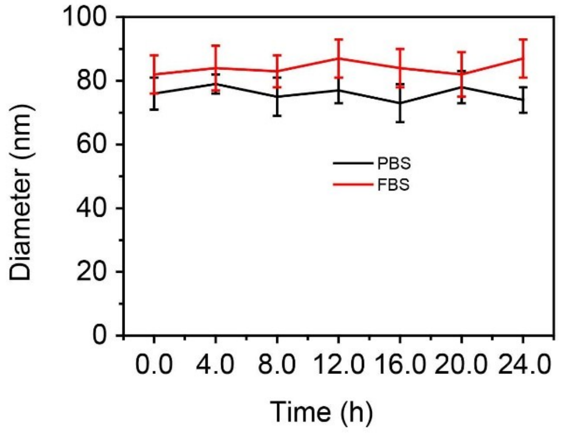

Figure S6. The change in DLS of CPPO@PEAZO-PCD NPs after storage under dark over time in PBS.

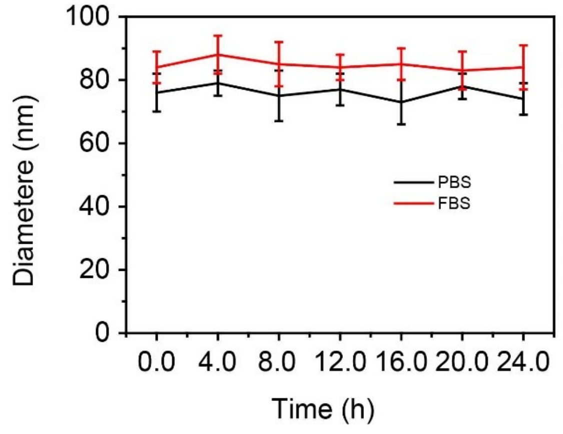

Figure S7. The change in DLS of CLDRSs after storage under dark over time in PBS.

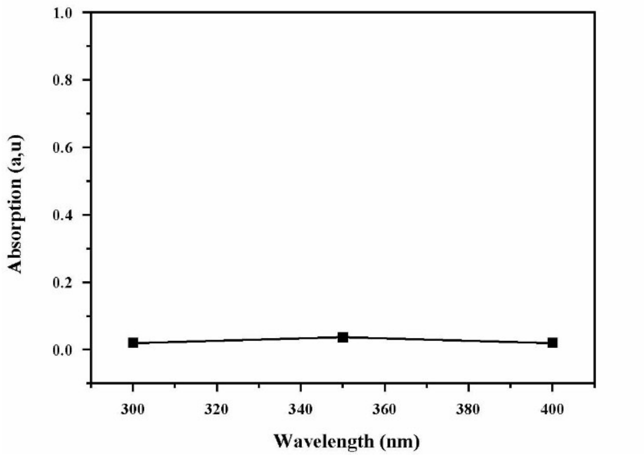

Figure S8. The UV-vis spectra of the PBS (pH = 7.4) solutions containing 2% Tween 20 (out of the dialysis bag suspended with CLDRSs incubated at 37 °C for 24 h. It was found that no obvious UV-vis absorption signal of CPT was found in the PBS solutions, indicating the CPT cannot be released from the bag MWCO 3500 in the dark.

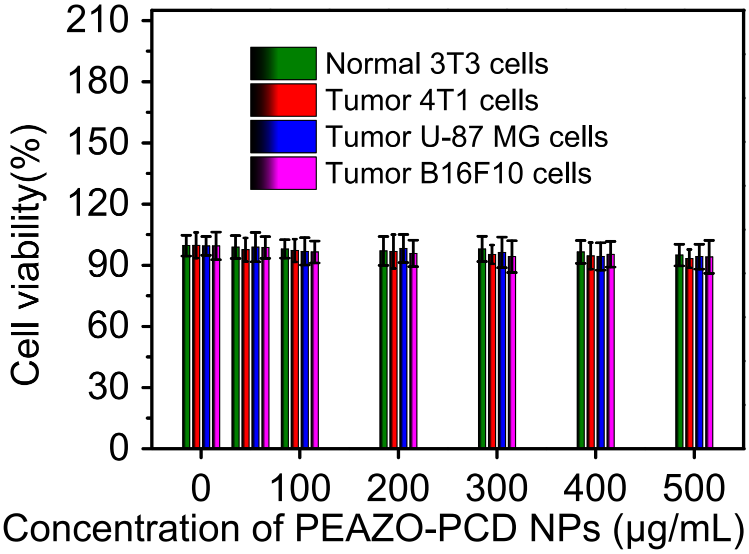

Figure S9. MTT assay for normal 3T3 cells and tumor cells (4T1, U-87 MG and B16F10 cells) treatment with PEAZO-PCD NPs at different concentrations. Data represent mean value ± standard deviation (n = 3).

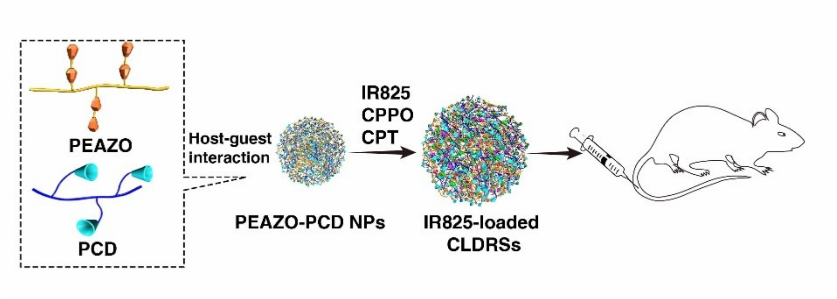

Figure S10. Schematic illustration of constructing IR825-loaded CLDRSs method and in vivo imaging of 4T1 tumor-bearing mice by tail vein injection of IR825-loaded CLDRSs.

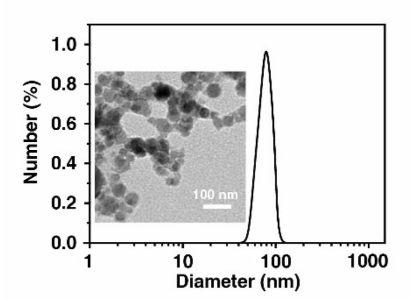

Figure S11. TEM and DLS of the CLDRSs.

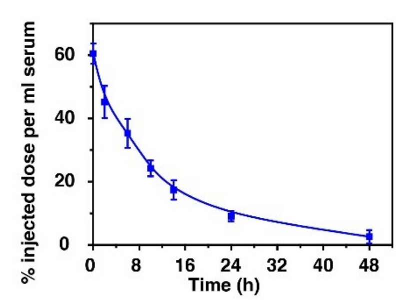

Figure S12. In vivo blood elimination kinetics of CLDRSs at a dose of 5 mg kg^{-1} body weight (n = 3 for each group).

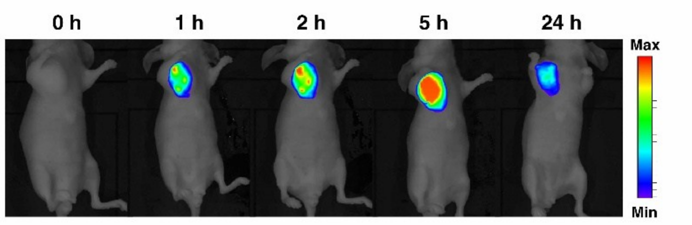

Figure S13. Fluorescence images (>800 nm) of tumor obtained after tail vein injection

of IR825-loaded CLDRSs (1 mg mL^{-1},100 μL).

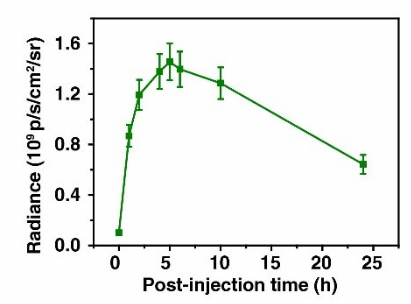

Figure S14. Integrated fluorescence intensity of tumor obtained after tail vein injection of IR825-loaded CLDRSs (1 mg mL^{-1},100 μL).

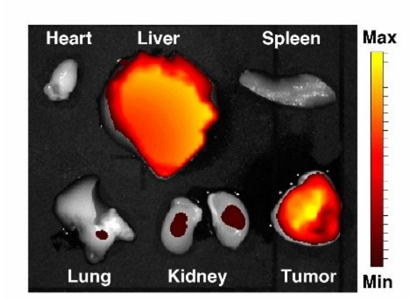

Figure S15. Fluorescence images of major organs and tumor obtained after tail vein injection of IR825-loaded CLDRSs for 5 h. Error bars indicate the s.d. (n = 3).

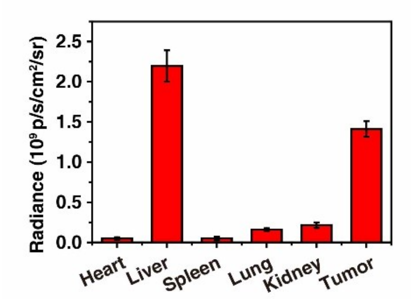

Figure S16. Integrated fluorescence intensity of major organs and tumor obtained after tail vein injection of IR825-loaded CLDRSs for 5 h. Error bars indicate the s.d. (n = 3).

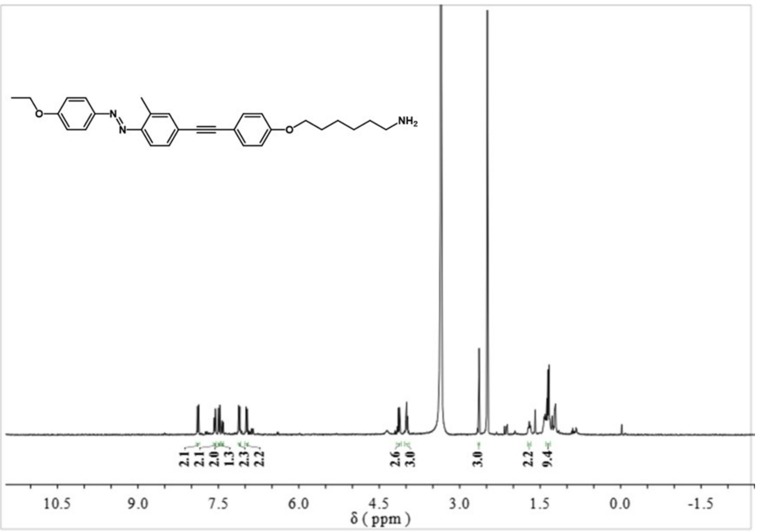

Figure S17. ^{1}H-NMR spectra of (E)-6-(4-((4-((4-ethoxyphenyl)diazenyl)-3-methylphenyl)ethynyl)phenoxy)hexan-1-amine (7) in DMSO-d_6.

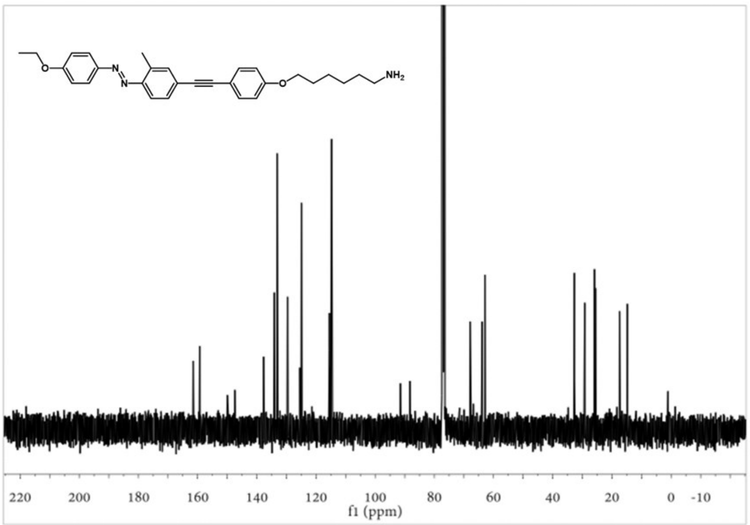

Figure S18. ^{13}C-NMR spectra of (E)-6-(4-((4-((4-ethoxyphenyl)diazenyl)-3-methylphenyl)ethynyl)phenoxy)hexan-1-amine (7) in DMSO-d_6.

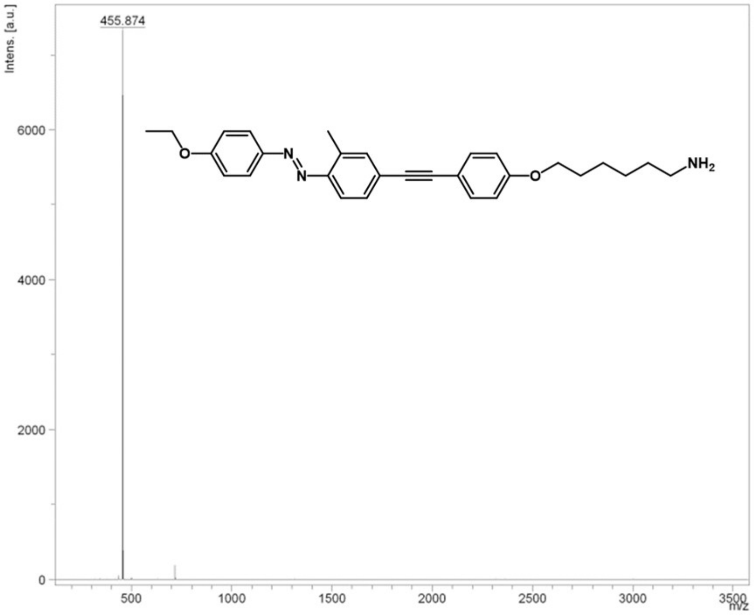

Figure S19. Matrix assisted laser desorption/ionization time-of-flight (MALDI-TOF) mass spectrum of (E)-6-(4-((4-((4-ethoxyphenyl)diazenyl)-3-methylphenyl)ethynyl)phenoxy)hexan-1-amine (7)**.**

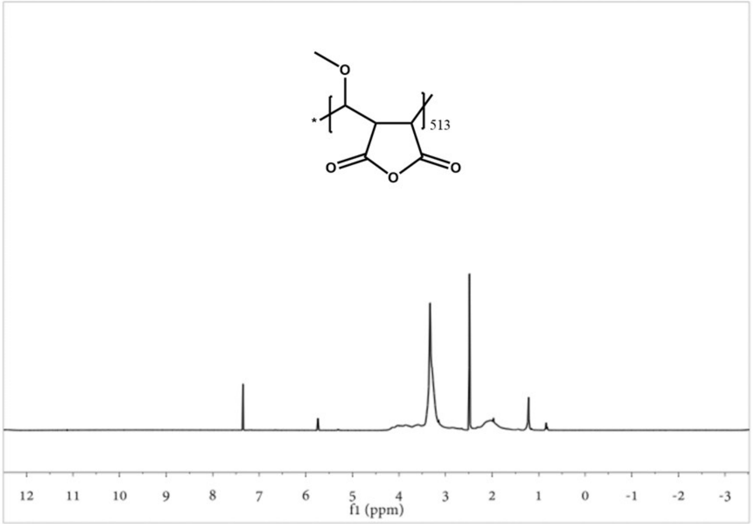

Figure S20. ^{1}H-NMR spectra of poly(methyl vinyl ether-alt-maleic anhydride) in DMSO-d_6.

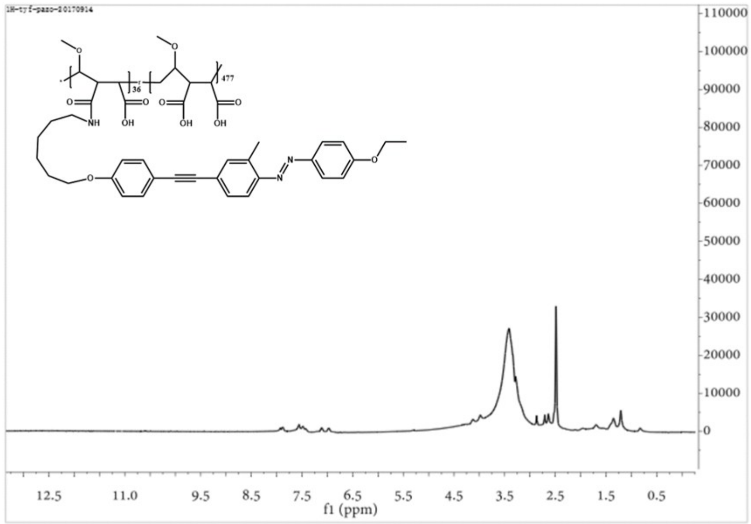

Figure S21. ^{1}H-NMR spectra of **PEAZO** in DMSO-d_6.

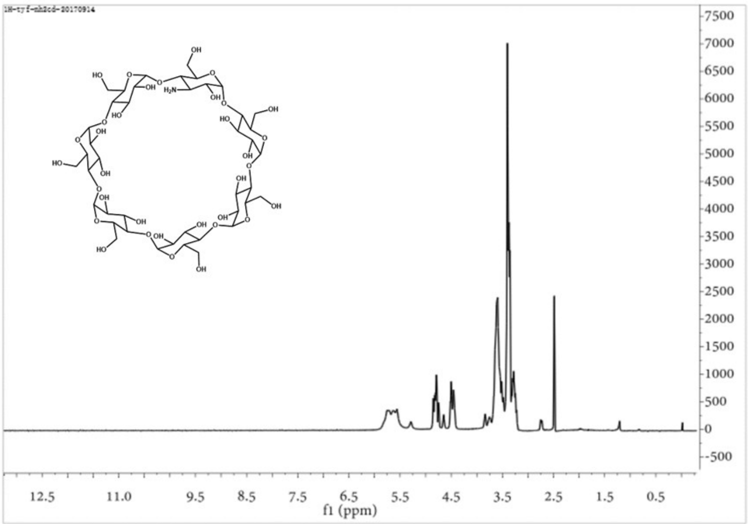

Figure S22. ^{1}H-NMR spectra of β-CD-NH_2 in DMSO-d_6.

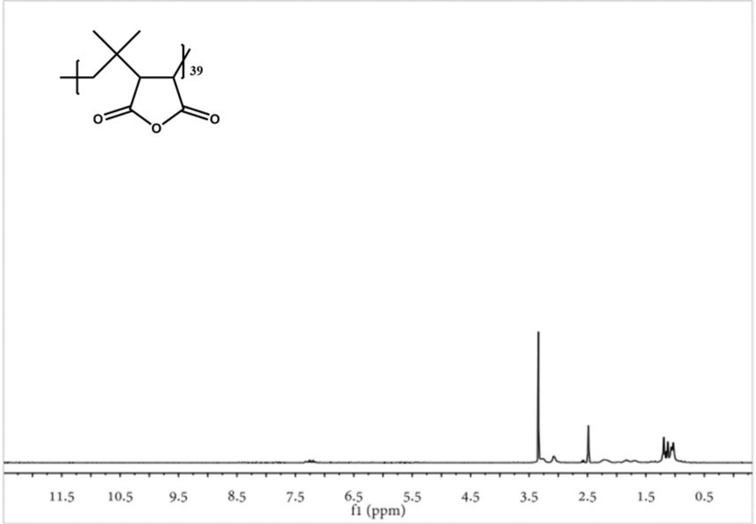

Figure S23. ^{1}H-NMR spectra of poly(isobutylene-alt-maleic anhydride) in DMSO-d_6.

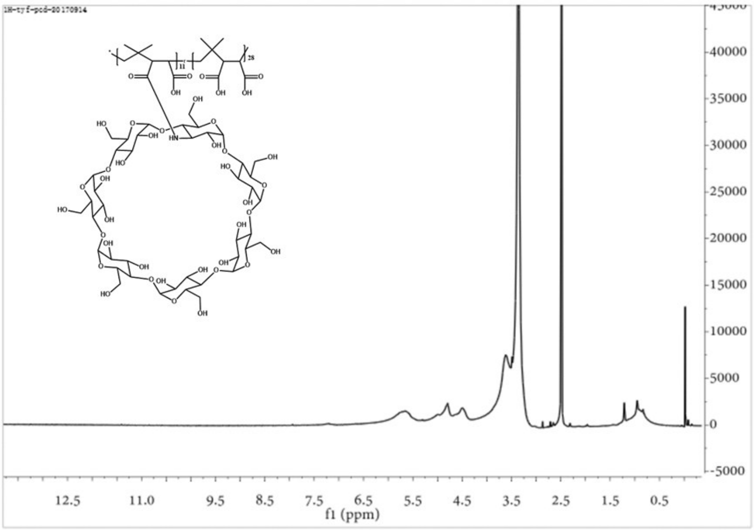

Figure S24. ^{1}H-NMR spectra of PCD in DMSO-d$_6$.

1. K. Okano, A. Shishido and T. Ikeda, Macromolecules, 2006, 39, 145-152.
2. D. Jańczewski, N. Tomczak, Y. W. Khin, M.-Y. Han and G. Julius Vancso, Eur. Polym. J., 2009, **45**, 3.
3. F. Cheng, Y. Zhang, R. Yin and Y. Yu, J. Mater. Chem., 2010, 20, 4888.
4. Z. Jiang, M. Xu, F. Li and Y. Yu, J. Am. Chem. Soc., 2013, **135**, 16446.
5. G. Yu, Z. Yang, X. Fu, B. C. Yung, J. Yang, Z. Mao, L. Shao, B. Hua, Y. Liu, F. Zhang, Q. Fan, S. Wang, O. Jacobson, A. Jin, C. Gao, X. Tang, F. Huang and X. Chen, Nat. commun., 2018, **9**, 766.
6. G. Yu, X. Zhao, J. Zhou, Z. Mao, X. Huang, Z. Wang, B. Hua, Y. Liu, F. Zhang, Z. He, O. Jacobson, C. Gao, W. Wang, C. Yu, X. Zhu, F. Huang and X. Chen, J. Am. Chem. Soc., 2018, **140**, 8005.
7. T. Sun, Q. Wang, Y. Bi, X. Chen, L. Liu, C. Ruan, Z. Zhao and C. Jiang, J. Mater. Chem. B, 2017, **5**, 2644.
